# The evolution of the aquaporin gene family and drought tolerance mechanisms in green plants

**DOI:** 10.1093/hr/uhaf209

**Published:** 2025-08-11

**Authors:** Yin Li, Shiqi Wen, Zihan Li, Rongrong Liu, Zhitong Zhang, Yan Li, Dianqiu Lyu, Hongju Jian

**Affiliations:** Integrative Science Center of Germplasm Creation in Western China (CHONGQING) Science City, Southwest University, Beibei, Chongqing 400715, China; College of Agronomy and Biotechnology, Southwest University, Beibei, Chongqing 400715, China; Key Laboratory of Germplasm Innovation of Upper Yangtze River, Ministry of Agriculture and Rural Affairs; Chongqing Key Laboratory of Biology and Genetic Breeding for Tuber and Root Crops, Chongqing 400715, China; Integrative Science Center of Germplasm Creation in Western China (CHONGQING) Science City, Southwest University, Beibei, Chongqing 400715, China; College of Agronomy and Biotechnology, Southwest University, Beibei, Chongqing 400715, China; Key Laboratory of Germplasm Innovation of Upper Yangtze River, Ministry of Agriculture and Rural Affairs; Chongqing Key Laboratory of Biology and Genetic Breeding for Tuber and Root Crops, Chongqing 400715, China; Integrative Science Center of Germplasm Creation in Western China (CHONGQING) Science City, Southwest University, Beibei, Chongqing 400715, China; College of Agronomy and Biotechnology, Southwest University, Beibei, Chongqing 400715, China; Key Laboratory of Germplasm Innovation of Upper Yangtze River, Ministry of Agriculture and Rural Affairs; Chongqing Key Laboratory of Biology and Genetic Breeding for Tuber and Root Crops, Chongqing 400715, China; Integrative Science Center of Germplasm Creation in Western China (CHONGQING) Science City, Southwest University, Beibei, Chongqing 400715, China; College of Agronomy and Biotechnology, Southwest University, Beibei, Chongqing 400715, China; Key Laboratory of Germplasm Innovation of Upper Yangtze River, Ministry of Agriculture and Rural Affairs; Chongqing Key Laboratory of Biology and Genetic Breeding for Tuber and Root Crops, Chongqing 400715, China; Integrative Science Center of Germplasm Creation in Western China (CHONGQING) Science City, Southwest University, Beibei, Chongqing 400715, China; College of Agronomy and Biotechnology, Southwest University, Beibei, Chongqing 400715, China; Key Laboratory of Germplasm Innovation of Upper Yangtze River, Ministry of Agriculture and Rural Affairs; Chongqing Key Laboratory of Biology and Genetic Breeding for Tuber and Root Crops, Chongqing 400715, China; Integrative Science Center of Germplasm Creation in Western China (CHONGQING) Science City, Southwest University, Beibei, Chongqing 400715, China; College of Agronomy and Biotechnology, Southwest University, Beibei, Chongqing 400715, China; Key Laboratory of Germplasm Innovation of Upper Yangtze River, Ministry of Agriculture and Rural Affairs; Chongqing Key Laboratory of Biology and Genetic Breeding for Tuber and Root Crops, Chongqing 400715, China; Integrative Science Center of Germplasm Creation in Western China (CHONGQING) Science City, Southwest University, Beibei, Chongqing 400715, China; College of Agronomy and Biotechnology, Southwest University, Beibei, Chongqing 400715, China; Key Laboratory of Germplasm Innovation of Upper Yangtze River, Ministry of Agriculture and Rural Affairs; Chongqing Key Laboratory of Biology and Genetic Breeding for Tuber and Root Crops, Chongqing 400715, China; Integrative Science Center of Germplasm Creation in Western China (CHONGQING) Science City, Southwest University, Beibei, Chongqing 400715, China; College of Agronomy and Biotechnology, Southwest University, Beibei, Chongqing 400715, China; Key Laboratory of Germplasm Innovation of Upper Yangtze River, Ministry of Agriculture and Rural Affairs; Chongqing Key Laboratory of Biology and Genetic Breeding for Tuber and Root Crops, Chongqing 400715, China

## Abstract

Aquaporins (AQPs) are integral membrane channel proteins that facilitate water transport and contribute significantly to plant adaptation under drought stress. However, the evolutionary origins and mechanisms of functional diversity of this gene family remain to be elucidated. A comprehensive genome-wide analysis was therefore performed on 104 representative species spanning the green plant lineage, from algae to angiosperms. This study used two datasets: Taxon I (algae to eudicots) and Taxon II (angiosperms including drought-tolerant and drought-sensitive plants). By systematically optimizing the gene structure, codon preferences, motifs, and cis-elements of these two datasets, the molecular mechanisms of AQP genes in plant adaptation evolution and drought-tolerance evolution were revealed. The results of phylogenetic analysis indicate that the AQP gene family is divided into five main subfamilies: PIPs, NIPs, TIPs, SIPs, and XIPs. Through in-depth analysis of the evolution characteristics of each subfamily, it was found that the emergence and loss of different subclusters are related to the ecological adaptation needs of specific species. By systematically analyzing the evolutionary history of the members of PIPs and TIPs subfamilies and subclusters, and combining their gene expression patterns, it was confirmed that PIP2, TIP1, and TIP4 subcluster members exhibit more significant expression response characteristics under drought stress. This study is the first to analyze the evolutionary patterns and drought-tolerance mechanisms of the AQP gene family at a multidimensional scale, providing important molecular targets for crop drought resistance breeding.

## Introduction

Aquaporins (AQPs), a family of small transmembrane proteins (21–32 kDa) characterized by six transmembrane α-helices, mediate their primary physiological functions through two highly conserved NPA (Asn–Pro–Ala) motifs. These proteins facilitate efficient transmembrane water transport via selective water channels, playing pivotal roles in plant growth and environmental stress responses [1]. Plant AQPs are systematically classified based on sequence homology and subcellular localization into currently recognizes eight subfamilies: GIPs, HIPs, LIPs, NIPs, PIPs, SIPs, TIPs, and XIPs [2]. Among these, NIPs, PIPs, SIPs, TIPs, and XIPs form the core AQPs components in green plants, while GIPs, LIPs, and HIPs are primarily found in prokaryotes and lower plants [3, 4]. Notably, GIPs pose challenges in phylogenetic owing to sequence similarities with the glycerol facilitator protein GIpF.

Advances in plant genomic data availability have enabled substantial progress in systematic evolutionary studies of the AQP gene family. Existing genomic analyses indicate that AQPs have undergone substantial gene number expansion and functional diversification from lower algae to higher angiosperms. Phylogenetic analysis of AQPs in model plants, such as *Arabidopsis thaliana* (35), rice (33), and wheat (113) has revealed distinct evolutionary trajectories between monocots and dicots [5–8]. Notably, comparative evolutionary studies between Solanaceae and Brassicaceae plants have clarified the phylogenetic relationships of specific subfamilies, confirming the conservative characteristics of this gene family in the adaptive evolution of terrestrial plants [9, 10]. However, the origin sequence and evolutionary divers of different AQPs subtypes remain unresolved, and the adaptive evolutionary mechanisms in extreme-environment species have yet to be elucidated.

As a major threat to global agriculture, drought stress has driven the evolution of multilayered adaptive mechanisms involving AQPs regulation. Research indicates that PIPs and TIPs, the primary water channels in plasma and vacuolar membranes, respectively, play central roles in drought response by regulating transmembrane water transport [11, 12]. Functional validation experiments confirm that heterologous expression of *BnPIP1*, *CfPIP2;1,* and *RhPIP2;1* significantly enhances drought-tolerance in host plants, underscoring the potential of AQPs for genetic improvement of drought resistance [13–15].

Despite substantial progress in functional characterization, the phylogenetic relationships among AQPs remain debated, with insufficient resolution of cross-taxa evolutionary patterns. This study systematically investigates the evolutionary history and drought adaptation mechanisms of AQPs across 104 green plant genomes, representing algae, bryophytes, lycophytes, ferns, gymnosperms, and angiosperms. Through cross-lineage phylogenetic reconstruction, we specifically address: (i) The origin of different subfamilies and subclusters; (ii) The evolutionary mechanism of AQP gene family and its association with terrestrial adaptability and drought tolerance; (iii) Signatures of adaptive evolution in drought-responsive subtypes (e.g. PIPs/TIPs). Our findings will provide a theoretical framework for elucidating the molecular mechanisms underlying environmental adaptation in plant AQPs, as well as crop improvement strategies.

## Results

### Distribution and evolutionary analysis of AQPs in green plants

**Figure 1:**
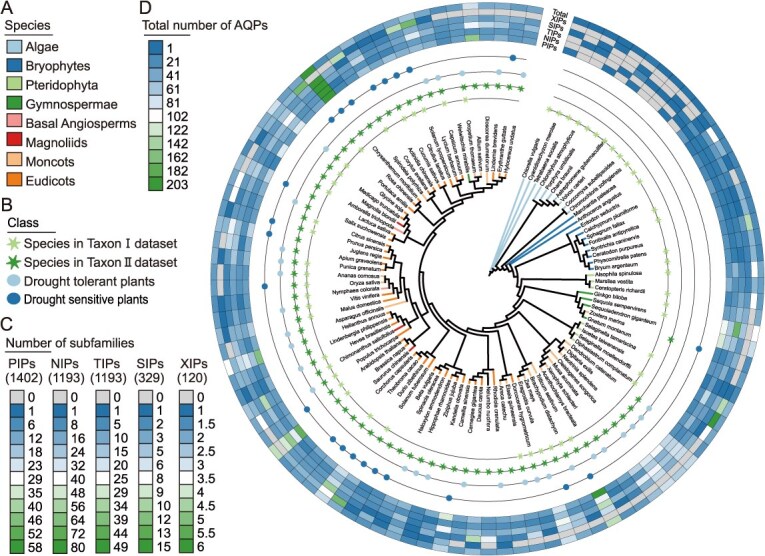
Evolutionary analysis of the aquaporin (AQP) gene family in 104 plant species. (A) A species phylogenetic tree spanning across algae, bryophytes, pteridophyta, gymnospermae, magnoliids, moncots, and eudicots, with evolutionary tree branches color coded according to taxonomic groups. (B) Species classification: Star represents dataset; The circle represents drought adapted species. (C) Distribution of offspring families. The heatmap ring (from inside to outside) displays the counts of PIPs, NIPs, TIPs, SIPs, and XIPs subfamilies (below the legend: subfamily names and total counts of each subfamily). (D) The total number of AQPs genes (outer circle) for each species.

To explore the evolutionary history of the AQP gene family, we performed a systematic comparative analysis of 104 representative plant species, including algae (10 species), bryophyta (10 species), pteridophyta (7 species), gymnospemae (5 species), and angiospermae (72 species). Using homology searches and domain prediction analyses, we identified 4237 putative AQPs proteins (Fig. 1A; Tables S1 and S2), confirming that AQPs subfamilies originated in algae and subsequently diversified [16]. A marked evolutionary expansion in gene copy number was observed, ranging from early-diverging algae (e.g. *Cyanidioschyzon merolae* with a single copy) to advanced eudicots (e.g. *Chrysanthemum morifolium* with 203 copies), reflecting progressive genomic expansion paralleling plant complexity (Fig. S1A; Table S1). Bryophytes exhibited limited AQPs numbers (16–23 copies), likely due to the absence of whole-genome duplication (WGD) events and their adaptation to humid environments. Lycophytes and ferns, a phylogenetically intermediate group occupying transitional niches, showed increased AQPs counts (20–52 copies), potentially associated with vascular system development. Gymnosperms (26–104 copies) and angiosperms (22–203 copies) underwent substantial AQPs proliferation, correlating with enhanced water regulation mechanisms and polyploidization events (Fig. S1A; Table S1). Notably, angiosperms accounted for 90% of the Top 20 species with the highest AQPs counts (9 eudicots, 7 monocots, 2 magnoliids), where elevated copy numbers (e.g. in *Chrysanthemum morifolium*, *Triticum aestivum*, and *Brassica napus*) exhibited strong correlations with genome expansion via polyploidization (Fig. S1B). To further investigate whether the number of AQPs in plants is related to the expansion of genome size and the number of encoded protein genes in corresponding species, correlation analysis revealed a positive correlation between the number of AQPs genes and the genome size and the number of encoded protein genes in corresponding plant species (Fig. S1C and D).

To better understand the relationship between the number of AQP gene subfamily and evolutionary adaptation, subfamily classification revealed that higher plants generally possessed PIPs (1402), NIPs (1193), TIPs (1193), SIPs (329), and XIPs (120) (Fig. 1C; Table S1). This was consistent with previous studies that identified eight AQPs subfamilies in early-diverging plant lineages. Apart from algal intrinsic proteins (LIPs), bryophyte GlpF-like intrinsic proteins (GIPs), and hybrid intrinsic proteins (HIPs), the remaining five subfamilies—PIPs, NIPs, TIPs, SIPs, and XIPs—exhibited widespread occurrence in higher plants and substantial diversification into phylogenetically distinct subgroups [3]. Furthermore, Taxon-specific dominance patterns were observed: algae (48.15%) and the magnoliid clade (38.36%) were predominantly composed of TIPs, while bryophytes (39.06%), gymnosperms (31.83%), monocots (34.77%), and eudicots (33.72%) were mainly dominated by PIPs. Lycophytes and ferns (46.95%) exhibited a preference for NIPs (Table S3). These findings reflected adaptive divergence in water management strategies across taxa.

To assess the evolutionary rates variation among across different taxonomic groups and determine whether natural selection contributed to genetic variation in the 4237 gene sequences, we performed pairwise *Ka/Ks* (the ratio of nonsynonymous to synonymous substitution rates) analyses on 4237 AQPs homologs from 104 species, stratified by phylogenetic lineage. Evolutionary dynamics analyses revealed strong purifying selection across the AQP gene family (*Ka/Ks* < 1), indicating functional conservation (Fig. S2). Except for the algal group, although bryophytes and lycophytes have experienced more nonsynonymous mutations (higher *Ka* values), they still mainly maintain functional stability (Table S4).

Gene duplication is recognized as a key driver of biological evolution, and analyzing duplication events provides deeper insights into the expansion mechanisms of the plant AQPs family. Gene duplication analysis identified five types of duplication events: single-copy (SL), dispersed segmental duplication (DSD), proximal duplication (PD), tandem duplication (TD), and whole-genome duplication (WGD) (Table S5). Among them, DSD dominated as the primary expansion mechanism across all lineages except gymnosperms (63.96%–93.62%), where SL exhibited the highest proportion (52.30%). This suggests that gymnosperms maintain gene expression homeostasis by retaining single-copy genes, consistent with patterns of purifying selection. WGD was restricted to the lycophyte-ferns, magnoliids, monocots, and eudicots, with the highest contribution in monocots (21.70%), potentially facilitating rapid adaptation to environmental stress (Fig. S2; Table S5). These results systematically reveal that the AQP gene family's expansion through multiple pathways while preserving functional conservation, thereby promoting terrestrial adaptation and ecological diversification in plants.

### Evolutionary features of AQPs gene structure

To systematically investigate the evolutionary characteristics of AQP genes from lower algae to higher plants, we classified them into Taxon I (1952 genes) and Taxon II (3467 genes) using drought classification system [17], Taxon II species (*n* = 72) were stratified into 30 drought-tolerant (DT), 24 drought-sensitive (DS), and 18 unclassified groups (Fig. 1B; Tables S6 and S7).

Based on the Taxon I dataset, we analyzed the evolutionary mechanisms of AQPs gene structure across plant lineages (Fig. S4A and B). The results showed that the number of exons in AQPs genes mainly ranges from 1 to 5 across the transition from bryophytes to higher plants. Early land plants exhibited significant structural differentiation: bryophytes had a high proportion of genes with six exons (16.20%), markedly exceeding that of other groups (<5%), and there was a significant difference in exon number compared with lycophytes and ferns (*P* < 0.01, Kruskal–Wallis test) (Table S8). The average total exons length in these two plant groups was 1444 and 1243 bp (Table S9), respectively suggesting that structural complexity (exon expansion) contributed to enhanced water regulation during terrestrial adaptation (Table S4). Notably, gymnosperms with larger genomes (4.11–26.5 Gb) showed a distinct evolutionary pattern: 47.65% of AQP genes retained three exons, with the short average exon length (883 bp). This structural simplification may contribute to maintaining gene expression stability, consistent with selective pressure revealed by the *Ka/Ks* analysis (Tables S8 and S9).

Analysis of the Taxon II dataset to investigate the evolutionary mechanisms of AQPs gene structure in angiosperms (Fig. S4C and D), the results showed that the number of exons in angiosperm AQPs genes was concentrated between 3 and 5 (66.4%–87.2%), indicating evolutionary conservation (Table S10). The average exon length in eudicots was the longest (1115.87 bp), but significantly shortened to 737.3 bp in the early-diverging magnoliids clade (*P* < 0.001, Kruskal–Wallis test), reflecting that gene function was optimized through structural simplification to enhance environmental adaptability (Table S11). Monocots have the lowest average number of exons (3.50), the highest *Ka* value (0.294), and the lowest *Ks* value (2.961), implying that they may have maintained genomic stability while enhancing adaptive potential through conservative selection (Table S4).

**Figure 2:**
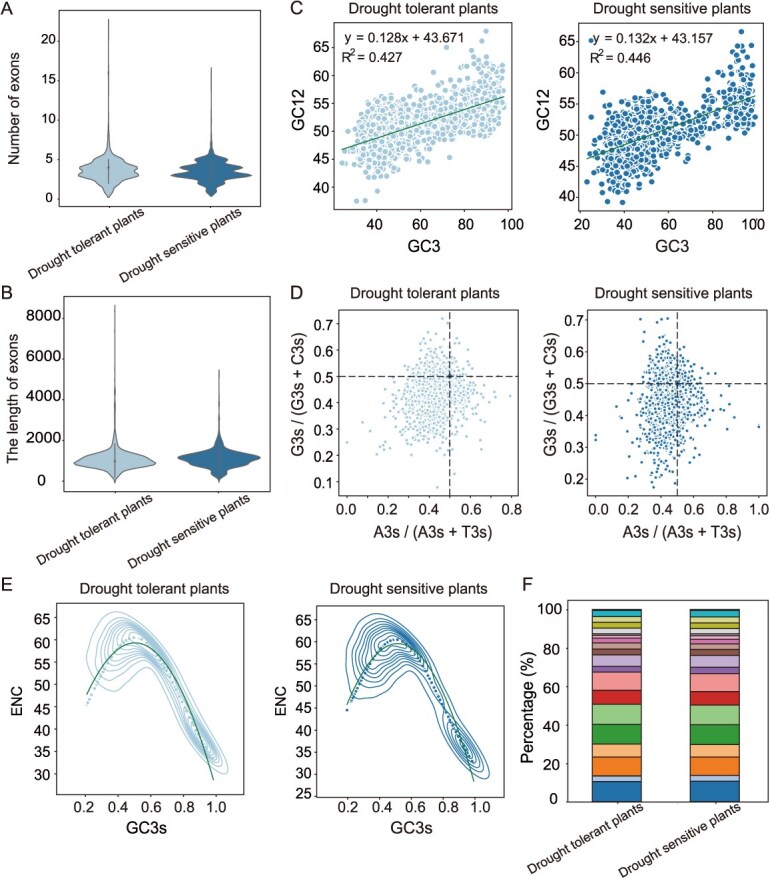
Analysis of the evolutionary mechanisms of AQPs genes in drought-sensitive and drought-tolerant plants. (A) Exon quantity analysis. (B) Exon length analysis. (C) Neutral plot analysis of codon preference. (D) Password preference PR2 plot analysis. (E) Password preference ENC plot analysis. (F) Analysis of the proportion of 20 feature motifs.

The AQP genes in both DT and DS plants were predominantly composed of 2 to 5 exons (89.94% vs 91.91%), but DT plants exhibited a significantly higher proportion of genes with 4 to 5 exons (46.18% vs 43.29%) and shorter average exon lengths (1071.18 vs 1105.64 bp) (Fig. 2A and B; Tables S12 and S13). This structural characteristic suggested that DT plants enhanced splicing diversity by increasing exons numbers while improving regulatory efficiency through exon shortening, thereby rapidly activating water balance-related genes under drought stress. This finding established a direct correlation between gene structural characteristics (exon number/length) and enhanced drought tolerance in plants.

### Evolutionary mechanisms of codon usage bias in AQPs genes

Codon usage bias regulates the translation process of genes, influencing their expression levels and translational efficiency, thereby shaping phenotypic traits and organismal adaptability [18]. Analysis of the Taxon I dataset revealed that AQPs genes exhibited significant GC bias at the third synonymous codon position (GC3s; mean, 58.34%), following the order C3s > G3s > T3s > A3s, with monocots showing the highest GC3s (83.13%) and gymnosperms the lowest (43.57%) (Table S14). Neutrality plot analysis identified natural selection as the dominant evolutionary driver (*R*^2^ = 0.094–0.345), with gymnosperms exhibiting the strongest influence of mutational pressure (*R*^2^ = 0.345) (Fig. S5A; Table S15). PR2 plot analysis demonstrated that genes in all groups except gymnosperms clustered in the third quadrant, indicating strong negative selection pressure, as supported by *Ka/Ks* analysis (Fig. S6A; Table S4). ENC plot further validated the predominant role of natural selection in shaping AQPs evolution across plant lineages, from lower algae to higher plants (Fig. S7A).

The analysis of Taxon II elucidated the evolutionary mechanisms of codon usage bias in angiosperm AQPs. The third base of synonymous codons exhibited a significant GC preference (mean 59.62%), following the order G3s > C3s > T3s > A3s, with monocots displaying the highest (74.70%) and dicots the lowest (47.56%) (Table S16). Mutational pressure contributed 11.8% to 41.9% (*R*^2^ = 0.118–0.419) to codon usage variation in Taxon II angiosperms (Fig. S5B; Table S17). The PR2 plot for the entire group was concentrated in the third quadrant, consistent with low *Ka/Ks* ratios and ENC plot results, collectively confirming strong negative selection as the dominant evolutionary force (Figs S6A and S7B; Table S4).

DT plants exhibit a stronger GC preference (GC3 60.90% vs 56.14%) and higher mutation pressure (42.7% vs 44.6%) compared to DS plants (Fig. 2C–E; Tables S18 and S19). The GC12 content (51.47%) observed in DT plants, combined with strong negative selection pressure, indicated that DT plants enhance translation efficiency by optimizing codon usage, thereby maintaining gene expression stability under drought stress. This mechanism provided a molecular evolutionary framework for plant drought tolerance.

### Evolutionary patterns of AQPs motifs

A total of 20 conserved motifs were identified using the Taxon I and II datasets (Fig. S8). Analysis of Taxon I showed that the NPA motif (motif 1 and 6) and the ar/R selectivity filter structure (motif 7 and 19) were identified as core functional elements of AQPs [19]. The highly conserved NPA motif was present in over 35% of plants outside algae (and over 50% in algae), maintaining water channel stability and selective transport functionality, while the proportion of the ar/R motif was less than 8% in all taxa, suggesting a potential function-specific activation mechanism (Table S20). The absence of motifs 13, 14, 15, and 18 in lower algae and motif 10 in bryophytes reflected the dynamic evolution of motifs related to terrestrial adaptation (Table S20). Subfamily-specific motif losses were prominent: PIPs lacked one motif (motif 18), TIPs lacked five motifs (2, 3, 13, 14, and 19), NIPs lacked five motifs (3, 7, 13, 14, and 19), SIPs lacked eight motifs (7, 9, 12, 13, 14, 15, 17, and 20), and XIPs lacked six motifs (2, 7, 13, 17, 18, and 19) (Table S21), reflecting distinct structural foundations underlying functional differentiation among subfamilies during evolution.

Analysis of the Taxon II dataset demonstrated high conservation of motifs in angiosperm AQPs, with seven core motifs (motifs 1, 4–8, and 10) constituting over 60% of total motifs and showing no lineage-specific losses (Table S22). Furthermore, DT plants exhibited significant upregulation of motifs 3, 4, 7, and 12 (+0.14%–0.17%) and downregulation of motif 14 (−0.21%) compared to DS plants, with these motifs implicated in membrane stability and aquaporin selectivity regulation (Fig. 2F; Table S23). These motif proportion adjustments may enhance water management efficiency in DT plants under drought stress by optimizing osmotic selectivity and structural stability of AQPs.

### Characteristics of AQPs cis-regulatory networks

Analysis of the promoter regions in the Taxon I and II datasets indicated that abiotic stress-responsive elements, particularly drought-related elements, were significantly enriched across all plant taxa (Fig. S9). In Taxon I, monocots exhibited the highest proportion of drought-related elements (37.67%), with key elements including ABRE (4.98%), MYB (4.61%), and TGACG-motif (3.20%), which aligned with their physiological adaptation to efficient water utilization (Tables S24 and S25). Gymnosperms exhibited the lowest proportion of drought elements (28.56%), which may be attributed to their unique drought regulation optimization strategy. In Taxon II, the proportion of drought elements in angiosperms is relatively stable (58.57%–63.13%), with monocots still showing enrichment of ABRE (7.66%) and TGACG-motif (5.28%), and a prominent proportion of DRE family elements (Tables S26 and S27).

DT plants exhibited higher total proportions of all four cis-regulatory element categories compared to DS plants (Table S28), with a significant increase in drought-related elements (33.18% vs 32.56%). Notably, core drought resistance elements showed marked enrichment in DT plants: TGACG motif (2.45% vs 2.09%), Myb (2.17% vs 1.84%), and MBS (1.09% vs 0.09%) (Table S29). This enhancement of regulatory networks may support DT plants' adaptive evolution in arid environments by strengthening the rapid activation capacity of stress-responsive genes, thereby providing key regulatory targets for deciphering molecular mechanisms of plant drought resistance.

### Phylogenetic evolution and functional diversification of AQPs subfamilies

**Figure 3:**
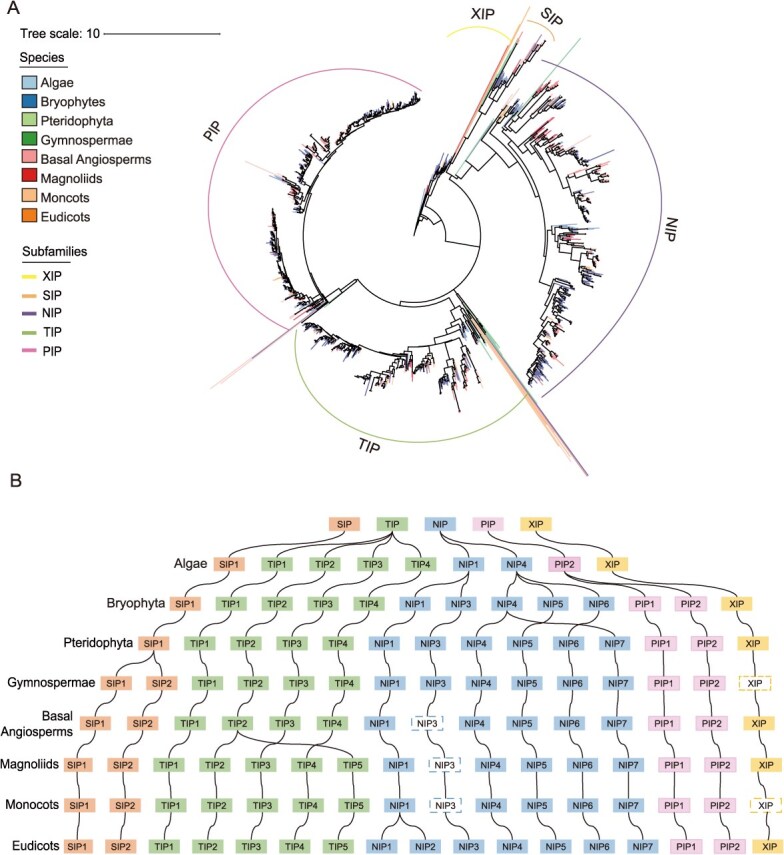
Phylogenetic and evolutionary trajectory analysis of AQP gene family. (A) Phylogenetic tree of AQPs gene subfamily. The legend ‘Species’ represents different species groups, corresponding to different branch colors in the evolutionary tree. The legend and ‘Subfamilies’ represents different subfamilies with different line colors. (B) The evolutionary trajectory of AQPs subfamily from lower algae to higher dicotyledonous plants. Different colored boxes represent different subfamilies. The dashed box represents the missing subfamily, and the curve represents the evolutionary path.

To elucidate the evolutionary trajectory of AQPs genes from lower algae to higher plants, we constructed a phylogenetic tree based on the Taxon I dataset. Phylogenetic topology analysis revealed that AQPs homologs are mainly classified into five major clades: SIPs, XIPs, NIPs, TIPs, and PIPs (Fig. 3A). SIPs and NIPs showed closer phylogenetic relationships with PIPs and TIPs, suggesting their possible origin from a common ancestor with subsequent divergence during evolution. Notably, XIP subfamily exhibited lineage-specific losses in gymnosperms, monocots, and Brassicaceae, indicating that the evolution of this subfamily is hindered in this group of species, and its function may have been replaced by some TIPs and NIPs with similar ar/R selective filters and transcriptional abundance profiles [20] (Fig. 3B). The remaining four subfamilies were conserved across eight evolutionary groups from algae to eudicots.

Further analysis of subcluster differentiation within the five subfamilies demonstrated that SIPs and PIPs diverged into SIP1/SIP2 and PIP1/PIP2 subclusters, respectively. NIPs and TIPs displayed the highest diversity: NIPs expanded from algal subclusters 1/4 to seven subclusters in eudicots, whereas TIPs evolved from algal subclusters 1–4 to five subclusters in eudicots (Fig. 3B). Specifically, NIP2;1 subcluster only appears in the Brassicaceae family, according to existing reports, Arabidopsis NIP2; 1 can regulate lactate efflux to regulate pyruvate metabolism [21]. The systematic absence of the NIP3;1 subcluster in basal plants, Magnoliaceae, and monocotyledonous species may be functionally compensated by other NIPs isoforms, particularly NIP1;1, NIP1;2, and NIP5;1, which exhibit overlapping substrate specificities including arsenite transport [22, 23].

Evolutionary tracing of TIPs subfamily suggested that the four algal subclusters likely contributed to vacuolar osmoregulation and stress adaptation, whereas diversification in higher plants aligned with functional demands of specialized vacuolar systems [3, 24]. For instance, TIP5;1 is a dedicated channel for transporting water and urea in pollen [25]. Phylogenetic evidence suggests that the TIP5 cluster evolved from TIP2 type aquaporins and maintains ancestral functions in the transport of ammonium ions (NH₄^+^) and ammonia (NH_3_) to vacuoles [26]. This evolutionary difference may have resulted in specialized adaptations to the unique transport requirements of pollen tubes during pollination, particularly for water, urea, and nitrogen-containing compounds.

**Figure 4:**
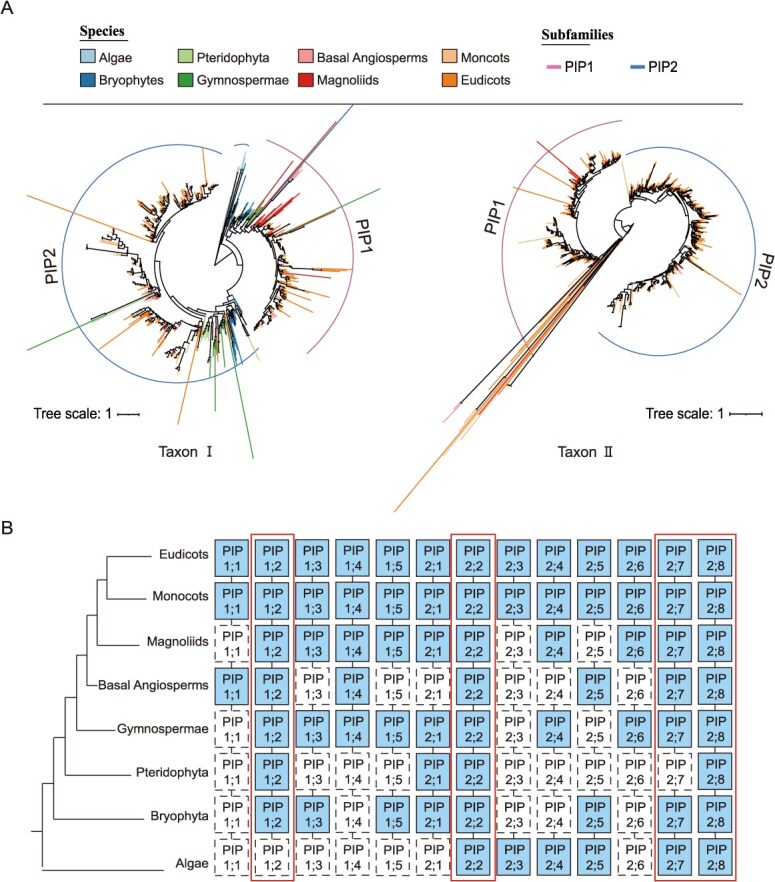
Phylogenetic tree and evolutionary trajectory of PIP subfamily. (A) Phylogenetic tree of PIPs genes in Taxon I and Taxon II datasets. The legend ‘Species’ represents different species groups, corresponding to different branch colors in the evolutionary tree. The legend ‘Subfamilies’ represents different subfamilies with different line colors. (B) The evolutionary trajectory of PIPs subclusters from lower algae to higher dicotyledonous plants. Solid squares represents present subclusters, dashed squares represents the missing subclusters, and large solid boxes represents subclusters that have either consistently existed or been missing only once during the evolution of PIPs.

**Figure 5:**
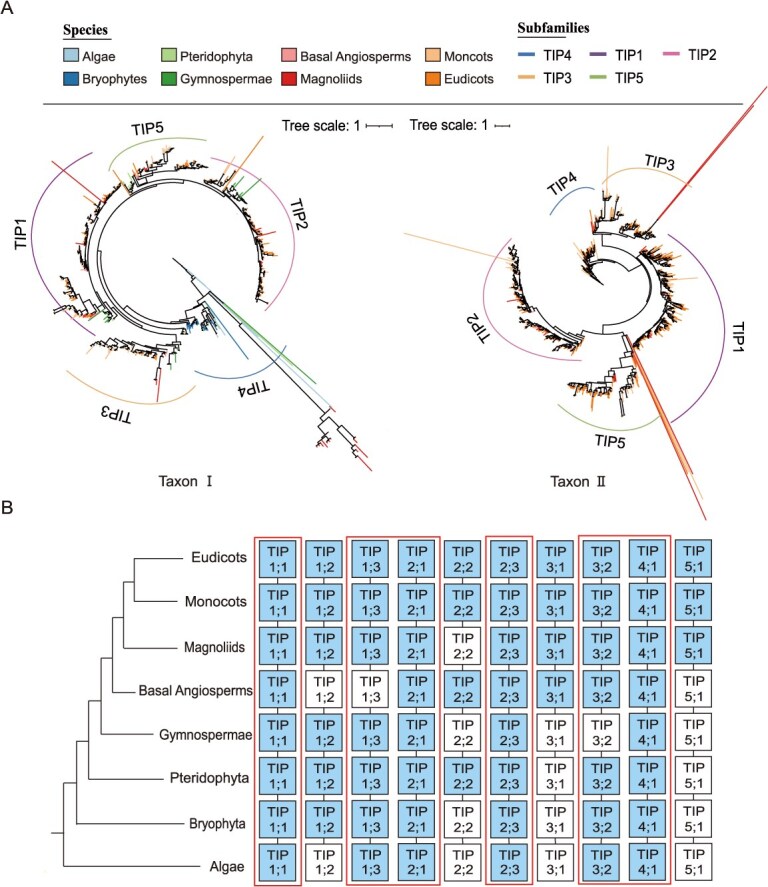
Phylogenetic tree and evolutionary trajectory of the TIPs subfamily. (A) Phylogenetic tree of TIPs genes in the Taxon I and Taxon II datasets. The legend ‘Species’ represents different species groups, corresponding to different branch colors in the evolutionary tree. The legend ‘Subfamilies’ represents different subfamilies with different line colors. (B) Evolutionary trajectory of TIPs subclusters from lower algae to higher dicotyledonous plants. Solid squares represents present subclusters, dashed squares represents the missing subclusters, and large solid boxes represents subclusters that have either consistently existed or been missing only once during the evolution of TIPs.

PIPs (mediating transmembrane water transport) and TIPs (regulating intracellular water balance) play complementary roles in drought response [27]. To elucidate their evolutionary relationships, phylogenetic trees and evolutionary trajectory diagrams of the PIP and TIP subfamilies in different lineages of plants from the Taxon I and II datasets were constructed (Figs 4 and 5). Phylogenetic analysis showed that PIP2;2, PIP2;3, PIP2;4, PIP2;5, PIP2;7, and PIP2;8 constitute the most ancient members of the PIP2 subclade. While PIP2 subcluster already existed in algae, PIP1 subcluster emerged during terrestrial adaptation (Fig. 4A). During the process of algal colonization of land and evolution into bryophytes, the PIP2;2 subclade further evolved into the PIP2;1 subclade. PIP2;1 further evolved into the PIP1;2 and PIP1;4 subclades, with the PIP1;2 subclade subsequently evolving into the PIP1;3, PIP1;5, and PIP1;1 subclades, while PIP2;2 further evolved into the PIP2;6 subclade (Fig. 4B). Distinct from the PIPs subfamily, the TIPs subfamily had already diversified into multiple subclusters during the algal evolutionary period, with TIP4 representing the most ancestral lineage (Fig. 5A). Subsequent evolutionary differentiation was observed: TIP1;1 subclade gradually evolving into the TIP1;2 subclade, the TIP3;2 subclade gradually evolving into the TIP3;1 subclade, TIP2;1 evolving into the TIP5;1 subclade, and the TIP2;3 subclade evolving into the TIP2;2 subclade (Fig. 5B). Collectively, these results offer novel insights into the phylogeny and evolutionary trajectories of the PIPs and TIPs subfamilies. During the evolutionary process from lower algae to higher dicotyledonous plants, members of the PIPs subclades (PIP1;2, PIP2;2, PIP2;7 and PIP2;8) and TIP subclades (TIP1;1, TIP1;3, TIP2;1, TIP3;2, and TIP4;1) have been retained, or were temporarily absent at specific evolutionary stages (Figs 4B and 5B). Therefore, these genes likely hold important functional positions within the PIP and TIP subfamilies, playing key roles in regulating the transmembrane transport of water and solutes. Additionally, they contribute to maintaining cellular hydration, enhancing root water uptake, and playing significant roles in coping with drought stress.

PIPs and TIPs play distinct yet complementary roles in plant drought response through their unique protein topologies. Analysis of the protein topologies of PIPs and TIPs revealed that both contain six transmembrane domains and conserved NPA motifs, but PIPs have higher sequence conservation. The key sites (LE1/LE2/H2/H5) in the ar/R selectivity filter region exhibit marked subcluster-specific variations: In the PIP subfamily, the H2 site is predominantly occupied by tryptophan (W), whereas TIP subfamily members preferentially feature leucine (L) or valine (V) at this position. The H5 site is conserved as histidine (H) in PIPs but replaced by isoleucine (I) or valine (V) in TIPs, with the TIP5 subcluster showing strict conservation of valine (V) at H5. At the LE1 position, threonine (T) was predominant in PIPs, while alanine (A) or glycine (G) were characteristic of TIPs. The LE2 site is invariantly arginine (R) in PIPs but diversified to arginine (R), valine (V), or cysteine (C) in TIPs, with cysteine (C) serving as a unique marker for the TIP5 subcluster. These conserved H5 (V) and lineage-specific LE2 (C) signatures provide diagnostic criteria for TIP5 subcluster identification (Figs S10 and S11; Table S30).

Analysis of Froger's positions (P1–P5) in PIPs and TIPs subclusters revealed that P3 (alanine, A) and P5 (tryptophan, W) were the most conserved across all sites. The P1 position was occupied by glutamine (Q) or tyrosine (Y) in PIPs but remained fixed as threonine (T) in TIPs. Notably, PIP1 and PIP2 subclusters exhibited distinct patterns of variation at P1. When combined with P2 site characteristics—highly conserved leucine (L) in PIP1 compared to serine (S) or alanine (A) in PIP2 and TIPs—these features enabled reliable discrimination between PIP1 and PIP2 members (Figs S10 and S11; Table S30).

The coordinated evolution of ar/R motifs and Froger's positions likely modulated substrate selectivity, driving functional divergence between PIPs (short-term drought response) and TIPs (long-term water storage). This structural–functional adaptive evolution provided a molecular mechanistic basis for plant drought adaptation.

### Identification of drought-resistant functional genes in PIPs and TIPs

We analyzed the expression of PIPs and TIPs genes in various tissues and organs, including roots, stems, leaves, flowers, and fruits, in five species (Table S31). The results showed significant differences in the expression of TIPs and PIPs genes across different tissues, suggesting that they may perform distinct biological functions in each tissue. In drought-sensitive plant *Zea mays*, the expression of TIPs and PIPs genes is mainly concentrated in the roots and ears. In *Oryza sativa*, TIP genes are more involved in water absorption in the roots and regulation during seed development, while PIP genes show more prominent expression in the leaves and flowering stages (Fig. S12). In drought-tolerant plant *Populus trichocarpa*, genes in the PIPs subfamily, such as PIP2;7 and PIP2;8, and TIP subfamily gene TIP4;1, have higher expression levels in the xylem, roots, and phloem. In *Brachypodium distachyon*, TIPs and PIPs genes are expressed in the leaves, inflorescences, and some reproductive organs (such as stamens), with TIPs genes primarily playing a role during early seed development, while PIPs genes are more prominent during later stages of seed development (Fig. S13). Additionally, the expression levels of PIPs and TIPs genes in some other tissues are relatively low, suggesting that their roles in these tissues may be limited or regulated by specific physiological conditions. We believe that the differential expression of TIPs and PIPs genes across different tissues may work in coordination to participate in water metabolism and drought responses in plants.

**Figure 6:**
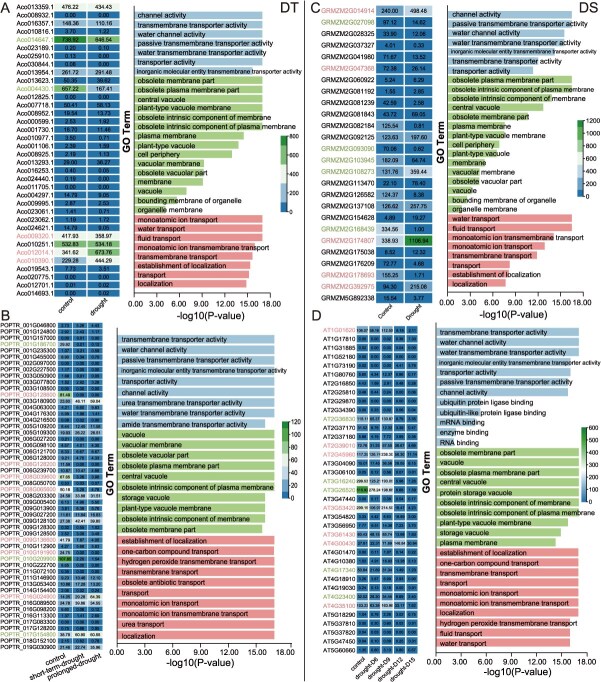
Expression patterns of PIPs/TIPs subfamily AQPs genes and GO enrichment analysis across plant species with contrasting drought tolerance. (A) Drought-tolerant *Ananas comosus*, (B) Drought-tolerant *Populus trichocarpa*, (C) Drought-sensitive *Arabidopsis thaliana*, and (D) Drought-sensitive *Zea mays*. In the heatmap, PIPs and TIPs subfamily genes responsive to drought stress are clearly labeled; in the GO enrichment analysis bar plots, different colors from top to bottom represent molecular functions, cellular components, and biological processes, respectively.

In this study, 17 representative angiosperms were selected to analyze the expression profiles of AQPs genes under drought stress (Table S31). The results revealed that among the five AQPs subfamilies, PIPs and TIPs subfamilies exhibited the most widespread drought-responsive characteristics, followed by NIPs and XIPs subfamilies. In-depth analysis of PIPs subclusters demonstrated significant differences in drought responsiveness: PIP2 subcluster members showed markedly higher response proportions than PIP1 subclusters, confirming PIP2’s core function in mediating transmembrane water transport [27, 28]. The drought-responsive characteristics of PIPs subclusters exhibit pronounced divergence in DT plants, with PIP2;1 and PIP2;2 subclusters demonstrating the most robust drought responsiveness. Specifically, PIP2;1 subcluster shows significant downregulation in pineapple (*Ananas comosus*) and black cottonwood (*Populus trichocarpa*), while PIP2;2 subcluster is upregulated in pineapple but downregulated in black cottonwood (Fig. 6A and B). In contrast, within the PIP subfamily of DS plants, PIP1;2, PIP1;4, and PIP2;2 subclusters universally participate in drought response, followed by secondary responders including PIP1;3, PIP2;4, PIP2;5, PIP2;7, and PIP2;8 subclusters. Notably, maize (*Zea mays*) displays the highest number of drought-responsive genes in the PIP2;2 subcluster, predominantly showing upregulated expression, whereas in *Arabidopsis thaliana*, most drought-responsive PIP genes are downregulated (Fig. 6C and D). Remarkably, PIP2;6 subcluster members remain unresponsive to drought across both DT and DS plants.

The drought-responsive patterns of TIPs subfamily reveal significant functional divergence: TIP1 and TIP4 subclusters broadly engage in drought response across plant species, followed by TIP2 and TIP3 subclusters, whereas TIP5 subcluster shows negligible responsiveness, suggesting its functional specialization may be governed by subcluster-specific regulation. Compared to DT plants, DS plants exhibit greater diversity in TIPs subclusters, with TIP1 subcluster consistently serving as the core drought-responsive module (Fig. 6A and D). Additionally, TIP4;1 subcluster in DS plants demonstrates distinct drought-responsive activity (Fig. 6C and D). GO enrichment analysis indicated that TIPs and PIPs genes primarily function in three biological dimensions: regulating transmembrane water transport at the molecular function level, localizing to vacuolar membranes at the cellular component level, and participating in nitrogen/ion transport at the biological process level (Fig. 6). This research systematically elucidates the critical roles of PIP2, TIP1, and TIP4 subclusters in plant drought resistance mechanisms, whose functional characteristics strongly correlate with the evolutionary trajectories of PIPs and TIPs subfamilies, providing a theoretical foundation for targeted exploration of drought-tolerant genes.

## Discussion

Plants have evolved a sophisticated water regulation network centered on AQPs, which play a pivotal role in drought stress responses by mediating transmembrane water transport. AQPs not only facilitate drought resistance by maintaining cellular osmotic balance but also dynamically modulate channel activity via signal transduction to enhance plant drought tolerance. However, the evolutionary origins and mechanisms underlying the functional divergence of the AQP gene family remain poorly understood. In this study, 4237 AQPs genes from 104 representative species (ranging from aquatic algae to higher angiosperms) to systematically investigate their evolutionary trajectory and molecular drought-resistance mechanisms, providing theoretical support for molecular breeding of drought-tolerant crops.

### Identification and classification of the AQP gene family and subfamilies

In the evolutionary analysis of AQP gene families, rational species selection and accurate gene identification are critical. Previous studies had demonstrated a robust framework for large-scale and systematic gene identification: PHGD integrated the genome annotations of 469 species, serving as a critical foundation for species selection [29]. Meanwhile, the PFGD and plantGIR databases offered extensive plant genomic resources and facilitate cross-species identification and extraction of homologous genes, making them particularly suitable for investigating the evolutionary conservation and divergence of gene families [30, 31]. Collectively, these databases enhanced both the systematic rigor and accuracy of this study.

As cross-species studies of AQP families advance, nomenclature inconsistencies leading to gene mis-annotation or omission have become increasingly apparent. For instance, this study revealed that the rice NIP5 subcluster was previously mislabeled as the NIP3 subcluster responsible for arsenate/antimonate transport in prior literature [32–34]. To establish a unified naming standard across species, we developed a standardized classification system based on phylogenetic analysis of *A. thaliana* AQPs orthologs.

### Origin and evolutionary expansion of the AQP gene family and subfamilies

Phylogenetic analysis indicates that the AQP gene family originated in algae and initially diverged into five major subfamilies: XIPs, SIPs, NIPs, TIPs, and PIPs (Figs 1C and 2A). Further analysis of the phylogenetic tree reveals the presence of transitional sequences between these five evolutionary branches, indicating that they may have undergone gene duplication and functional differentiation. For example, there are a small number of sequences of NIPs and TIPs between the XIPs and SIPs branches, which suggests that SIPs may have a common ancestor with NIPs and TIPs and are closely related to the ancestors of XIPs. In addition, partial PIPs sequences were also found between NIPs and TIPs branches, indicating that NIPs may share some ancestral features with PIPs and TIPs through convergent evolution or horizontal gene transfer. These findings provide important clues for understanding the evolutionary path and functional differentiation of the AQP gene family. Notably, XIPs underwent functional loss in gymnosperms and monocots, suggesting compensatory mechanisms by other subfamilies in these lineages. AQP gene numbers exhibited significant diversity during the evolutionary transition from non-vascular to vascular plants. The family underwent two major expansions: the first occurred during the terrestrial transition of algae to bryophytes, marked by a surge in gene numbers and functional diversification, providing molecular foundations for early land plants to adapt to fluctuating water availability; the second coincided with the emergence of seed plants, where gymnosperm AQPs expanded from ~20 to over 100 genes, driving functional specialization among subfamilies (Fig. 1D). Gene duplication analysis revealed that dispersed duplication is the primary mechanism for AQP family expansion. AQPs in crops such as wheat [8], rapeseed [35], and maize [36] predominantly arose via tandem or segmental duplication, indicating that both mechanisms jointly drive family expansion. Importantly, all AQPs across lineages were under strong purifying selection (*Ka/Ks* < 1), confirming the evolutionary conservation of their core water transport function, consistent with *Ka/Ks* analyses in wheat [8] and rapeseed [35].

While this study focuses on the five universal AQPs subfamilies in plants, the evolutionary origins of GIPs and HIPs require further investigation. Although GIPs and HIPs have been identified in non-vascular plants like *Physcomitrella patens* [3] and *Selaginella moellendorffii* [37], their absence in flowering plants may result from functional redundancy. Studies suggest GIPs originated from ancestral bacterial genes and were transferred to land plants alongside PIPs during algal evolution, with all three subfamilies potentially sharing an algal ancestor [19, 38]. However, GIPs/HIPs were selectively lost during angiosperm evolution.

### Multidimensional analysis of AQPs evolutionary adaptation mechanisms

Plant AQP exon–intron structures are highly conserved. This study found that exon numbers across species predominantly range from 1 to 5 (Table S8), consistent with findings in *Olea europaea* OeuAQPs [39], *Gossypium raimondii* GrAQPs [40] and *Betula pendula* BpeAQPs [41]. Motif analysis revealed high similarity in composition and arrangement within the same AQP subfamily, suggesting functional conservation across homologous subfamilies. As observed in *Olea europaea* OeuAQPs [41], motif patterns differ significantly between subfamilies, while critical functional motifs (e.g. NPA motifs, ar/R selectivity filters, and Froger residues) remain evolutionarily conserved, likely linked to subfamily-specific functions. Substrate selectivity differences among subfamilies may arise from substantial divergence in ar/R filters [42, 43], aligning with our observation of low ar/R motif representation from algae to eudicots (Table S20) and their context-dependent activation. Cis-acting element analysis identified drought-responsive motifs (TGACG-motif, ABRE, ARE, MYB, MYC, and STRE) in AQP promoters across bryophytes to eudicots (Table S25), corroborating reports of gibberellin-, abscisic acid-, and auxin-responsive elements in *Populus euphratica* PeuAQP promoters [44].

### Evolutionary mechanisms of AQPs in drought-tolerant plants

Drought tolerance is a multifaceted trait governed by complex gene networks. As key drought-response components, AQPs enhance plant resilience through dynamic regulation [45]. Comparative analysis revealed significant differences between drought-tolerant (DT) and drought-sensitive (DS) plants: DT AQPs exhibit shorter exon lengths and higher G + C content (Tables S18 and S29). Building on rice studies showing that weakly expressed genes are longer with lower third-codon G + C content [46], we hypothesize that DT AQPs may exhibit stronger expression. Additionally, DT AQPs are enriched with drought-associated cis-elements (Table S29), providing regulatory advantages in arid environments. However, our drought classification covers limited species, and uneven DT/DS sampling may reduce statistical power, necessitating broader taxonomic inclusion for robust mechanistic insights.

This study systematically elucidates the evolutionary dynamics and drought-adaptive mechanisms of plant AQPs, identifying PIP2 and TIP1/4 subclusters as key targets for drought-tolerant crop engineering. Future research should integrate epigenetic regulation and protein interaction networks to fully unravel AQP-mediated adaptation under drought stress.

## Materials and methods

### AQP gene family identification and classification

AQP gene data were retrieved from Phytozome v13.0, Ensembl Plants, NCBI, and species-specific genome databases (Table S1). Candidate protein sequences were screened using the PF00230 domain from the Pfam database and HMMER v3.0 (*E*-value ≤ 1e−5), followed by refinement through BLAST v2.15.0 alignment against the *A. thaliana* AQP reference set. Domain integrity was validated using SMART and CDD databases, and splice variants were excluded to finalize AQPs members. Subfamily classification adhered to the *Arabidopsis* AQPs classification system, supplemented by sequence similarity analysis [6].

### Phylogenetic analysis

Orthologous genes were identified using Broccoli v1.2 [47]. Species-specific super-sequences were aligned with MAFFT v7.520 [48], and species trees were reconstructed using IQ-TREE v1.6.12 (LG + R10 model; 1000 bootstrap replicates) [49]. Phylogenetic analysis was performed separately for the five AQPs subfamilies using Taxon I protein sequences. Sequences shorter than 250 bp were first removed, followed by multiple sequence alignment using ClustalW v2.1 [50] and phylogenetic tree construction with IQ-TREE under the Bayesian model (1000 bootstrap replicates). For PIPs/TIPs subfamily analysis, Taxon I sequences were aligned using both MAFFT v7.520 and ClustalW v2.1 without length filtering, while Taxon II sequences underwent more stringent filtering (<200 bp removed) prior to ClustalW alignment and IQ-TREE analysis (1000 bootstraps). All trees were visualized using iTOL, with multiple sequence alignments rendered in Jalview v2.11.4 [51].

### Gene structure and functional element analysis

Exon counts, lengths, and lineage-specific differences were calculated from GFF/GFF3 annotations using Python. Normality test (Shapiro–Wilk test) was performed on the data, followed by the Kruskal–Wallis test using the Python statsmodels package, and *post hoc* multiple comparisons (Dunn test) were conducted using the scikit-posthocs package. Motif analysis was performed using MEME v5.5.2 [52] to identify 20 conserved motifs, with motif frequencies across taxa calculated using unique counts per sequence (repeated motifs counted once). Cis-regulatory elements in promoter regions (2000 bp upstream of transcription start sites) were analyzed using the PlantCare [53] database, focusing on 42 stress-responsive elements (e.g. ABRE, DRE, MYB/MYC binding sites), with distribution frequencies quantified.

### Codon usage bias evaluation

Codon usage analysis was performed using Python to calculate GC content (GC1/GC2/GC3) and CodonW v1.4.2 to determine codon usage parameters (CAI, RSCU, ENc, codon bias index, optimal codon frequency, hydrophobicity, aromaticity, and base composition [T3s/C3s/A3s/G3s/GC/GC3s]). Three analytical approaches were employed: (i) ENC plot analysis comparing observed vs theoretical ENc values (ENc = 2 + GC3s + 29/[GC3s^2^ + (1 − GC3s)^2^]); (ii) PR2 plot analysis with A3s/(A3s + T3s) vs G3s/(G3s + C3s); and (iii) neutrality plot analysis regressing GC12 against GC3 to assess mutation-selection balance.

### Gene evolution and expression regulation

Gene duplication types were identified using MCScanX, including single-copy (SL), dispersed segmental duplication (DSD), proximal duplication (PD), tandem duplication (TD), and whole-genome duplication (WGD). Synteny analysis was performed using ParaAT 2.0 [54] and KaKs_Calculator 2.0 [55] to calculate the Ka/Ks values of homologous gene pairs (alignment was conducted using MAFFT v7.520). The baseline expression data (FPKM values) of AQPs genes were obtained from the Expression Atlas database (https://www.ebi.ac.uk/gxa/home), which includes transcript-level analyses across various tissues and organs of the species. The drought stress expression profiles of AQPs were sourced from the PlantExp [56], GERDH [57], and NCBI GEO (https://www.ncbi.nlm.nih.gov/geo/) databases. Using these resources, we extracted the FPKM values of AQPs genes and visualized their expression patterns through heatmap analysis. For GO enrichment analysis, the genomic protein sequences were uploaded to the eggNOG-mapper v2 [58] database for annotation. Subsequently, the ‘eggNOG-mapper Helper’ function in the TBtools (v2.2) [59] software was used to process the eggNOG-mapper files from the eggNOG annotation results. The out.emapper.annotations.GO.txt file was used for GO enrichment analysis, and the out.emapper.annotations.KEGG_Knum.txt file was used as the background annotation file for KEGG enrichment analysis. PIPs and TIPs genes were selected for visualization.

### Data visualization

Statistical analyses and visualizations were implemented using the Python seaborn package. 

## Supplementary Material

Web_Material_uhaf209

## Data Availability

The authors confirm that the data supporting the findings of this study are available within the article.

## References

[1] Singh RK, Deshmukh R, Muthamilarasan M. et al. Versatile roles of aquaporin in physiological processes and stress tolerance in plants. Plant Physiol Bioch. 2020;149:178–89 10.1016/j.plaphy.2020.02.009 32078896

[2] Johanson U, Gustavsson S. A new subfamily of major intrinsic proteins in plants. Mol Biol Evol. 2002;19:456–61 11919287 10.1093/oxfordjournals.molbev.a004101

[3] Danielson JAH, Johanson U. Unexpected complexity of the Aquaporin gene family in the moss. BMC Plant Biol. 2008;8:45 18430224 10.1186/1471-2229-8-45 PMC2386804

[4] Deshmukh R, Bélanger RR. Molecular evolution of aquaporins and silicon influx in plants. Funct Ecol. 2016;30:1277–85

[5] Deshmukh RK, Sonah H, Bélanger RR. Plant aquaporins: genome-wide identification, transcriptomics, proteomics, and advanced analytical tools. Front Plant Sci. 2016;7:1896 28066459 10.3389/fpls.2016.01896 PMC5167727

[6] Johanson U, Karlsson M, Johansson I. et al. The complete set of genes encoding major intrinsic proteins in Arabidopsis provides a framework for a new nomenclature for major intrinsic proteins in plants. Plant Physiol. 2001;126:1358–69 11500536 10.1104/pp.126.4.1358 PMC117137

[7] Nguyen MX, Moon S, Jung KH. Genome-wide expression analysis of rice aquaporin genes and development of a functional gene network mediated by aquaporin expression in roots. Planta. 2013;238:669–81 23801298 10.1007/s00425-013-1918-9

[8] Madrid-Espinoza J, Brunel-Saldias N, Guerra FP. et al. Genome-wide identification and transcriptional regulation of aquaporin genes in bread wheat (*Triticum aestivum* L.) under water stress. Genes-Basel. 2018;9:497 30326657 10.3390/genes9100497 PMC6210132

[9] De Rosa A, Watson-Lazowski A, Evans JR. et al. Genome-wide identification and characterisation of Aquaporins in *Nicotiana tabacum* and their relationships with other *Solanaceae* species. BMC Plant Biol. 2020;20:266 32517797 10.1186/s12870-020-02412-5 PMC7285608

[10] Diehn TA, Pommerrenig B, Bernhardt N. et al. Genome-wide identification of aquaporin encoding genes in *Brassica oleracea* and their phylogenetic sequence comparison to *Brassica* crops and *Arabidopsis*. Front Plant Sci. 2015;6:166 25904922 10.3389/fpls.2015.00166 PMC4387931

[11] Sutka MR, Manzur ME, Vitali VA. et al. Evidence for the involvement of hydraulic root or shoot adjustments as mechanisms underlying water deficit tolerance in two genotypes. J Plant Physiol. 2016;192:13–20 26803215 10.1016/j.jplph.2016.01.002

[12] Prado K, Maurel C. Regulation of leaf hydraulics: from molecular to whole plant levels. Front Plant Sci. 2013;4:255 23874349 10.3389/fpls.2013.00255 PMC3711007

[13] Afzal Z, Howton T, Sun Y. et al. The roles of aquaporins in plant stress responses. J Dev Biol. 2016;4:9 29615577 10.3390/jdb4010009 PMC5831814

[14] Jang JY, Rhee JY, Kim DG. et al. Ectopic expression of a foreign aquaporin disrupts the natural expression patterns of endogenous aquaporin genes and alters plant responses to different stress conditions. Plant Cell Physiol. 2007;48:1331–9 17675323 10.1093/pcp/pcm101

[15] Zhang S, Feng M, Chen W. et al. In rose, transcription factor PTM balances growth and drought survival via PIP2;1 aquaporin. Nat Plants. 2019;5:290–9 30833710 10.1038/s41477-019-0376-1

[16] McGaughey SA, Tyerman SD, Byrt CS. An algal PIP-like aquaporin facilitates water transport and ionic conductance. BBA-Biomembranes. 2021;1863:183661 34058166 10.1016/j.bbamem.2021.183661

[17] Bowles AMC, Paps J, Bechtold U. Evolutionary origins of drought tolerance in spermatophytes. Front Plant Sci. 2021;12:655924 34239520 10.3389/fpls.2021.655924 PMC8258419

[18] Parvathy ST, Udayasuriyan V, Bhadana V. Codon usage bias. Mol Biol Rep. 2022;49:539–65 34822069 10.1007/s11033-021-06749-4 PMC8613526

[19] Maurel C, Boursiac Y, Luu DT. et al. Aquaporins in plants. Physiol Rev. 2015;95:1321–58 26336033 10.1152/physrev.00008.2015

[20] Gupta AB, Sankararamakrishnan R. Genome-wide analysis of major intrinsic proteins in the tree plant: characterization of XIP subfamily of aquaporins from evolutionary perspective. BMC Plant Biol. 2009;9:134 19930558 10.1186/1471-2229-9-134 PMC2789079

[21] Beamer ZG, Routray P, Choi WG. et al. Aquaporin family lactic acid channel NIP2;1 promotes plant survival under low oxygen stress in Arabidopsis. Plant Physiol. 2021;187:2262–78 34890456 10.1093/plphys/kiab196 PMC8644545

[22] Xu WZ, Dai W, Yan H. et al. *Arabidopsis* NIP3;1 plays an important role in arsenic uptake and root-to-shoot translocation under arsenite stress conditions. Mol Plant. 2015;8:722–33 25732589 10.1016/j.molp.2015.01.005

[23] Kamiya T, Tanaka M, Mitani N. et al. NIP1;1, an aquaporin homolog, determines the arsenite sensitivity of *Arabidopsis thaliana*. J Biol Chem. 2009;284:2114–20 19029297 10.1074/jbc.M806881200

[24] Jauh GY, Phillips TE, Rogers JC. Tonoplast intrinsic protein isoforms as markers for vacuolar functions. Plant Cell. 1999;11:1867–82 10521518 10.1105/tpc.11.10.1867 PMC144099

[25] Soto G, Alleva K, Mazzella MA. et al. *AtTIP1;3* and *AtTIP5;1*, the only highly expressed Arabidopsis pollen-specific aquaporins, transport water and urea. FEBS Lett. 2008;582:4077–82 19022253 10.1016/j.febslet.2008.11.002

[26] Loqué D, Ludewig U, Yuan LX. et al. Tonoplast intrinsic proteins AtTIP2;1 and AtTIP2;3 facilitate NH_3_ transport into the vacuole. Plant Physiol. 2005;137:671–80 15665250 10.1104/pp.104.051268 PMC1065367

[27] Heinen RB, Ye Q, Chaumont F. Role of aquaporins in leaf physiology. J Exp Bot. 2009;60:2971–85 19542196 10.1093/jxb/erp171

[28] Kapilan R, Vaziri M, Zwiazek JJ. Regulation of aquaporins in plants under stress. Biol Res. 2018;51:4 29338771 10.1186/s40659-018-0152-0 PMC5769316

[29] Feng S, Liu Z, Chen H. et al. PHGD: an integrative and user-friendly database for plant hormone-related genes. Imeta. 2024;3:e164 38868516 10.1002/imt2.164 PMC10989150

[30] Wu T, Liu Z, Yu T. et al. Flowering genes identification, network analysis, and database construction for 837 plants. Hortic Res. 2024;11:uhae013 38585015 10.1093/hr/uhae013 PMC10995624

[31] Liu Z, Zhang C, He J. et al. plantGIR: a genomic database of plants. Hortic Res. 2024;11:uhae342 39712867 10.1093/hr/uhae342 PMC11661351

[32] Bienert GP, Thorsen M, Schüssler MD. et al. A subgroup of plant aquaporins facilitate the bi-directional diffusion of As(OH)_3_ and Sb(OH)_3_ across membranes. BMC Biol. 2008;6:26 18544156 10.1186/1741-7007-6-26 PMC2442057

[33] Chen Y, Sun SK, Tang Z. et al. The Nodulin 26-like intrinsic membrane protein OsNIP3;2 is involved in arsenite uptake by lateral roots in rice. J Exp Bot. 2017;68:3007–16 28505352 10.1093/jxb/erx165

[34] Sun SK, Chen Y, Che J. et al. Decreasing arsenic accumulation in rice by overexpressing *OsNIP1;1* and *OsNIP3;3* through disrupting arsenite radial transport in roots. New Phytol. 2018;219:641–53 29749629 10.1111/nph.15190

[35] Kayum MA, Park JI, Nath UK. et al. Genome-wide expression profiling of aquaporin genes confer responses to abiotic and biotic stresses in *Brassica rapa*. BMC Plant Biol. 2017;17:23 28122509 10.1186/s12870-017-0979-5 PMC5264328

[36] Chaumont F, Barrieu F, Wojcik E. et al. Aquaporins constitute a large and highly divergent protein family in maize. Plant Physiol. 2001;125:1206–15 11244102 10.1104/pp.125.3.1206 PMC65601

[37] Anderberg HI, Kjellbom P, Johanson U. Annotation of major intrinsic proteins and the evolution of the protein family in terrestrial plants. Front Plant Sci. 2012;3:33 22639644 10.3389/fpls.2012.00033 PMC3355642

[38] Gustavsson S, Lebrun AS, Nordén K. et al. A novel plant major intrinsic protein in most similar to bacterial glycerol channels. Plant Physiol. 2005;139:287–95 16113222 10.1104/pp.105.063198 PMC1203378

[39] Li WX, Zhang DY, Zhu GZ. et al. Combining genome-wide and transcriptome-wide analyses reveal the evolutionary conservation and functional diversity of aquaporins in cotton. BMC Genomics. 2019;20:538 31262248 10.1186/s12864-019-5928-2 PMC6604486

[40] Venisse JS, Õunapuu-Pikas E, Dupont M. et al. Genome-wide identification, structure characterization, and expression pattern profiling of the aquaporin gene family in *Betula pendula*. Int J Mol Sci. 2021;22:7269 34298887 10.3390/ijms22147269 PMC8304918

[41] Faize M, Fumanal B, Luque F. et al. Genome wild analysis and molecular understanding of the aquaporin diversity in olive trees (*Olea europaea* L.). Int J Mol Sci. 2020;21:4183 32545387 10.3390/ijms21114183 PMC7312470

[42] Bansal A, Sankararamakrishnan R. Homology modeling of major intrinsic proteins in rice, maize and *Arabidopsis*: comparative analysis of transmembrane helix association and aromatic/arginine selectivity filters. BMC Struct Biol. 2007;7:27 17445256 10.1186/1472-6807-7-27 PMC1866351

[43] Wallace IS, Roberts DM. Homology modeling of representative subfamilies of Arabidopsis major intrinsic proteins. Classification based on the aromatic/arginine selectivity filter. Plant Physiol. 2004;135:1059–68 15181215 10.1104/pp.103.033415 PMC514140

[44] Ndayambaza B, Si J, Zhou D. et al. Genome-wide analysis of aquaporins gene family in *Populus euphratica* and its expression patterns in response to drought, salt stress, and phytohormones. Int J Mol Sci. 2024;25:10185 39337672 10.3390/ijms251810185 PMC11432731

[45] Haghpanah M, Hashemipetroudi S, Arzani A. et al. Drought tolerance in plants: physiological and molecular responses. Plants-Basel. 2024;13:2962 39519881 10.3390/plants13212962 PMC11548289

[46] Camiolo S, Melito S, Porceddu A. New insights into the interplay between codon bias determinants in plants. DNA Res. 2015;22:461–70 26546225 10.1093/dnares/dsv027 PMC4675714

[47] Derelle R, Philippe H, Colbourne JK. Broccoli: combining phylogenetic and network analyses for orthology assignment. Mol Biol Evol. 2020;37:3389–96 32602888 10.1093/molbev/msaa159

[48] Katoh K, Standley DM. MAFFT multiple sequence alignment software version 7: improvements in performance and usability. Mol Biol Evol. 2013;30:772–80 23329690 10.1093/molbev/mst010 PMC3603318

[49] Nguyen LT, Schmidt HA, von Haeseler A. et al. IQ-TREE: a fast and effective stochastic algorithm for estimating maximum-likelihood phylogenies. Mol Biol Evol. 2015;32:268–74 25371430 10.1093/molbev/msu300 PMC4271533

[50] Thompson JD, Higgins DG, Gibson TJ. Clustal-W: improving the sensitivity of progressive multiple sequence alignment through sequence weighting, position-specific gap penalties and weight matrix choice. Nucleic Acids Res. 1994;22:4673–80 7984417 10.1093/nar/22.22.4673 PMC308517

[51] Waterhouse AM, Procter JB, Martin DMA. et al. Jalview version 2-a multiple sequence alignment editor and analysis workbench. Bioinformatics. 2009;25:1189–91 19151095 10.1093/bioinformatics/btp033 PMC2672624

[52] Bailey TL, Boden M, Buske FA. et al. MEME SUITE: tools for motif discovery and searching. Nucleic Acids Res. 2009;37:W202–8 19458158 10.1093/nar/gkp335 PMC2703892

[53] Lescot M, Déhais P, Thijs G. et al. PlantCARE, a database of plant cis-acting regulatory elements and a portal to tools for in silico analysis of promoter sequences. Nucleic Acids Res. 2002;30:325–7 11752327 10.1093/nar/30.1.325 PMC99092

[54] Zhang Z, Xiao J, Wu J. et al. ParaAT: a parallel tool for constructing multiple protein-coding DNA alignments. Biochem Biophys Res Commun. 2012;419:779–81 22390928 10.1016/j.bbrc.2012.02.101

[55] Wang D, Zhang Y, Zhang Z. et al. KaKs_Calculator 2.0: a toolkit incorporating gamma-series methods and sliding window strategies. Genom Proteom Bioinform. 2010;8:77–80 10.1016/S1672-0229(10)60008-3 PMC5054116 20451164

[56] Liu JD, Zhang Y, Zheng Y. et al. PlantExp: a platform for exploration of gene expression and alternative splicing based on public plant RNA-seq samples. Nucleic Acids Res. 2023;51:D1483–91 36271793 10.1093/nar/gkac917 PMC9825497

[57] Cheng H, Zhang H, Song J. et al. GERDH: an interactive multi-omics database for cross-species data mining in horticultural crops. Plant J. 2023;116:1018–29 37310261 10.1111/tpj.16350

[58] Cantalapiedra CP, Hernández-Plaza A, Letunic I. et al. eggNOG-mapper v2: functional annotation, orthology assignments, and domain prediction at the metagenomic scale. Mol Biol Evol. 2021;38:5825–9 34597405 10.1093/molbev/msab293 PMC8662613

[59] Chen CJ, Wu Y, Li J. et al. TBtools-II: a “one for all, all for one” bioinformatics platform for biological big-data mining. Mol Plant. 2023;16:1733–42 37740491 10.1016/j.molp.2023.09.010

