# Supplementary figures and images for: The evolution of the aquaporin gene family and drought tolerance mechanisms in green plants
Source: Hortic Res. 2025 Aug 11;12(11):uhaf209. doi: 10.1093/hr/uhaf209 (PMC12578469; doi:10.1093/hr/uhaf209)

A

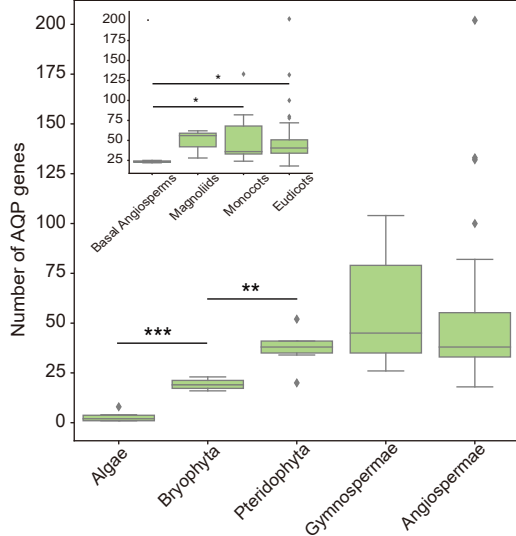

B

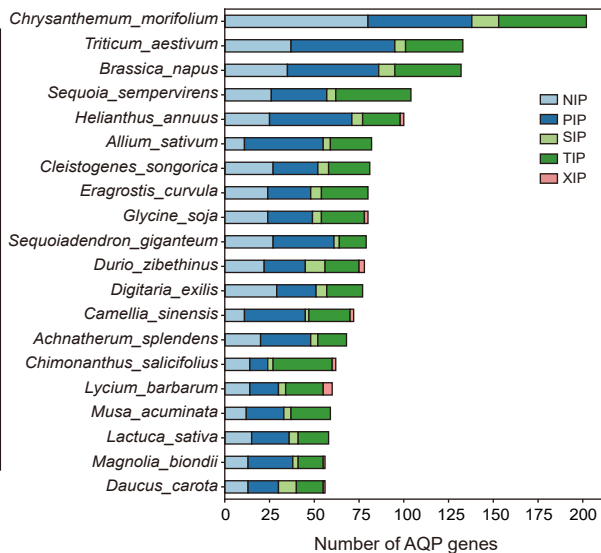

C

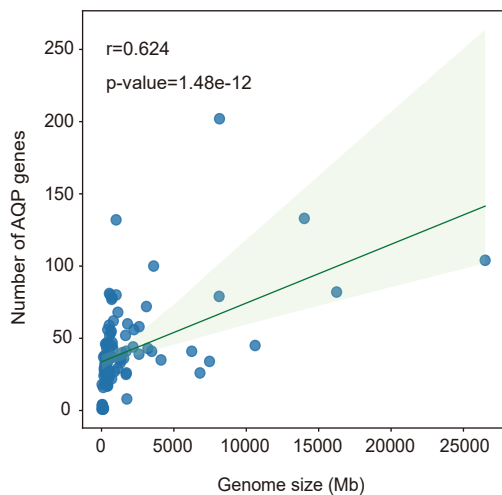

D

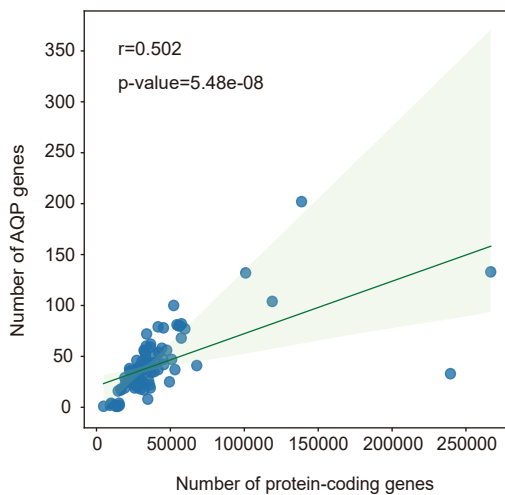

Supplement: Web_Material_uhaf209 [file web_material_uhaf209.zip › Figure S1.pdf]

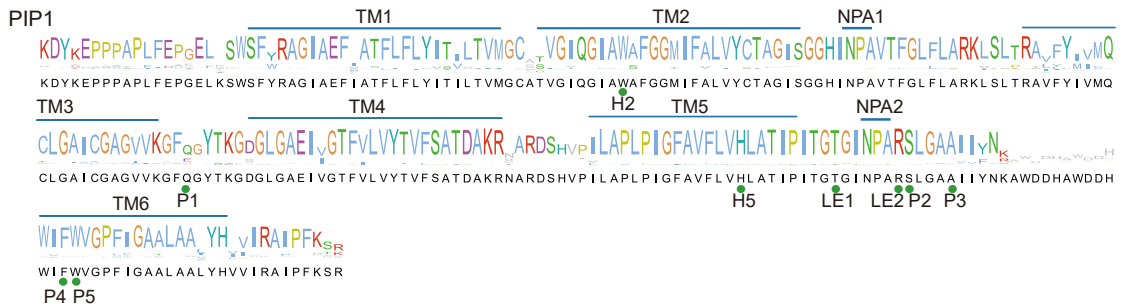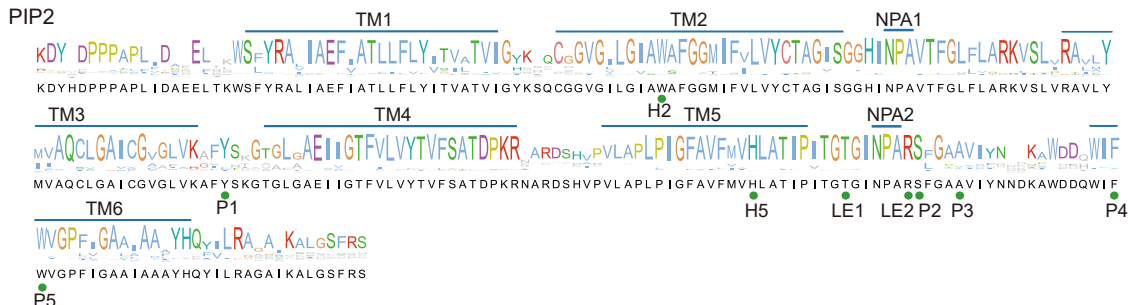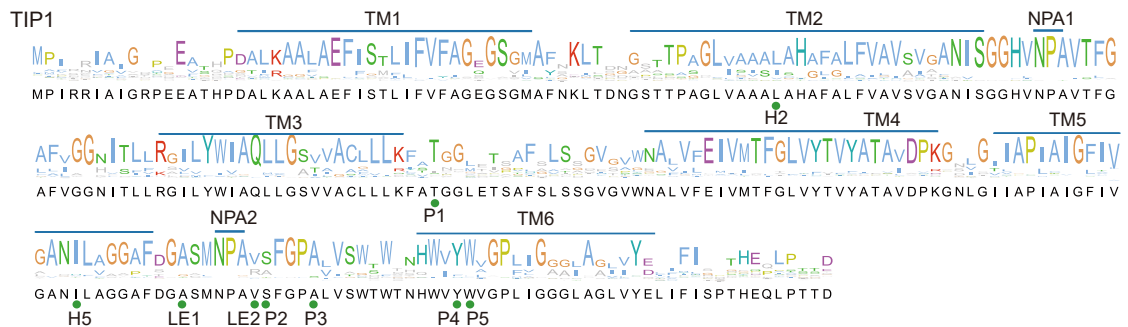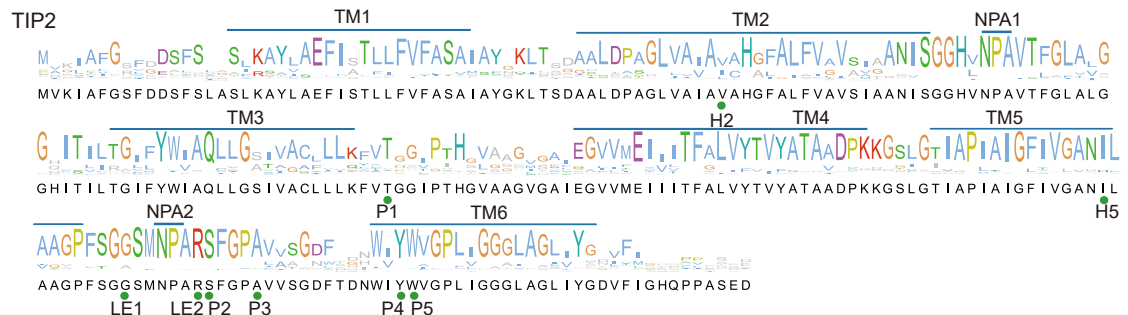

Supplement: Web_Material_uhaf209 [file web_material_uhaf209.zip › Figure S10.pdf]

## TIP3

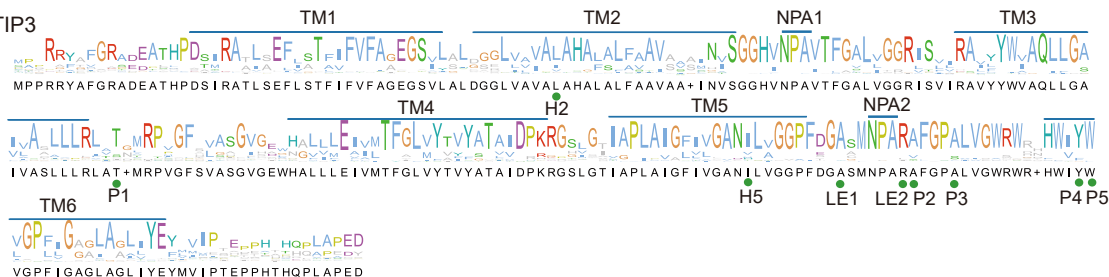

## TIP4

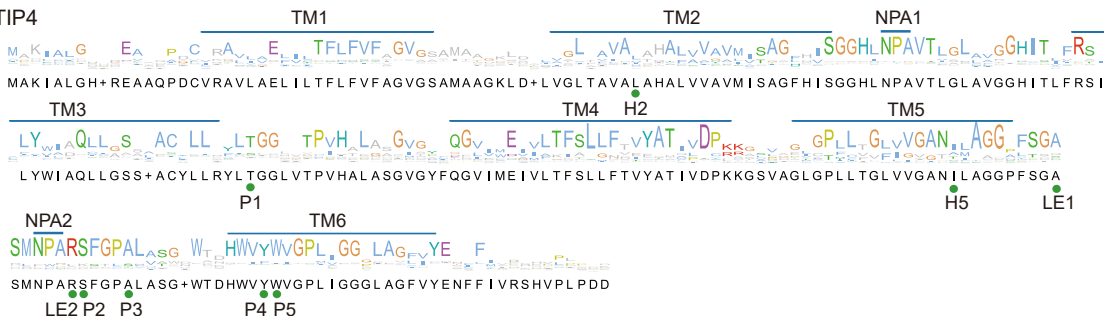

## TIP5

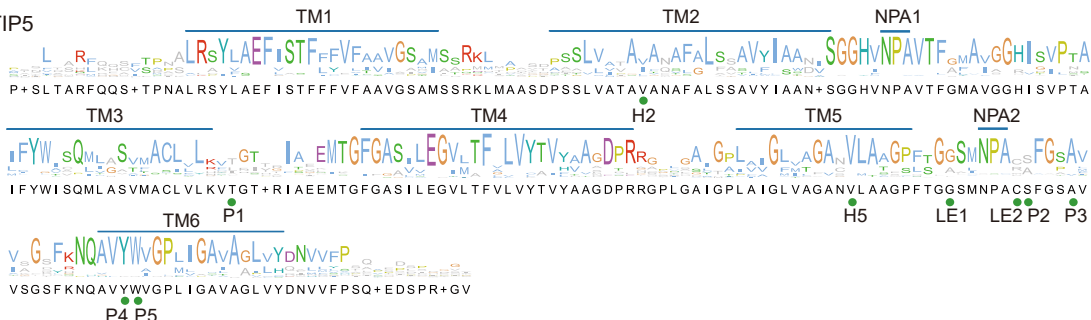

Supplement: Web_Material_uhaf209 [file web_material_uhaf209.zip › Figure S11.pdf]

DS

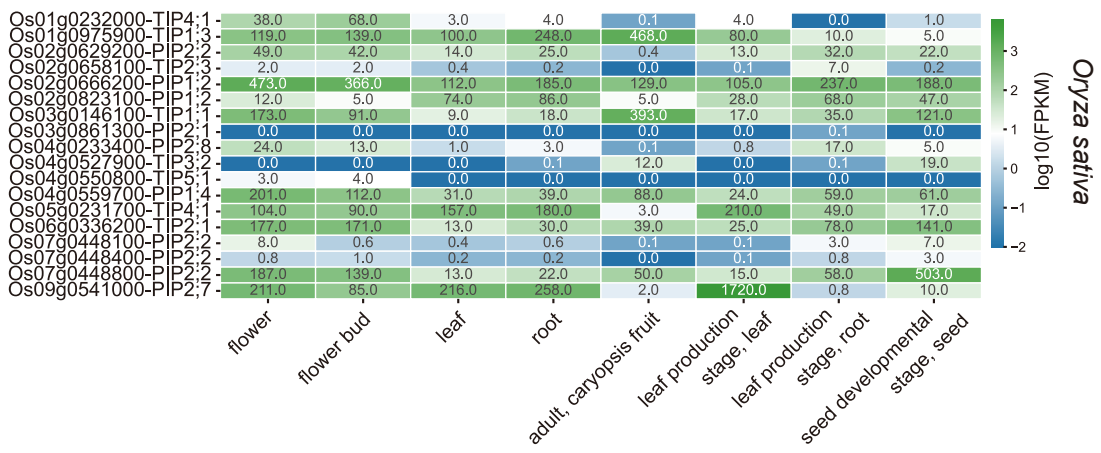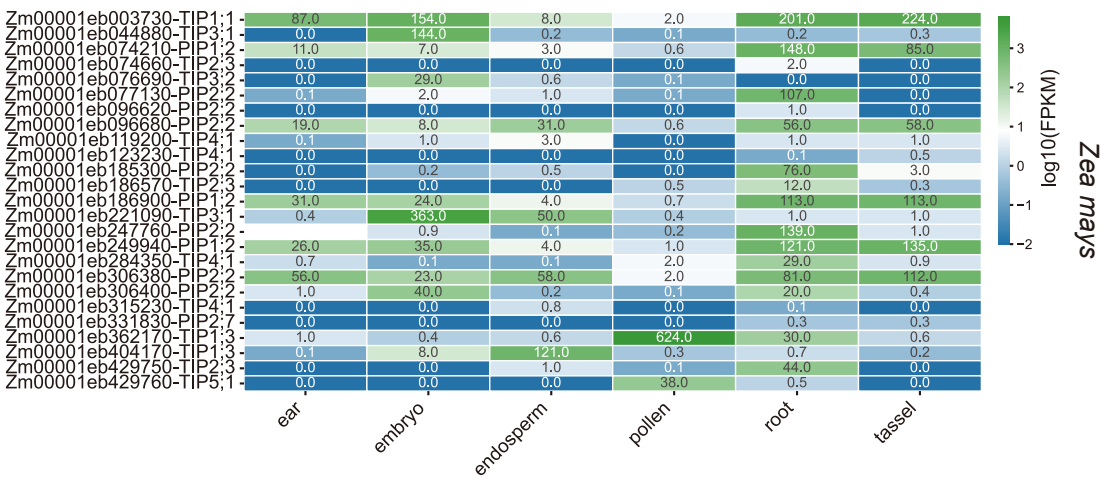

Supplement: Web_Material_uhaf209 [file web_material_uhaf209.zip › Figure S12.pdf]

DS

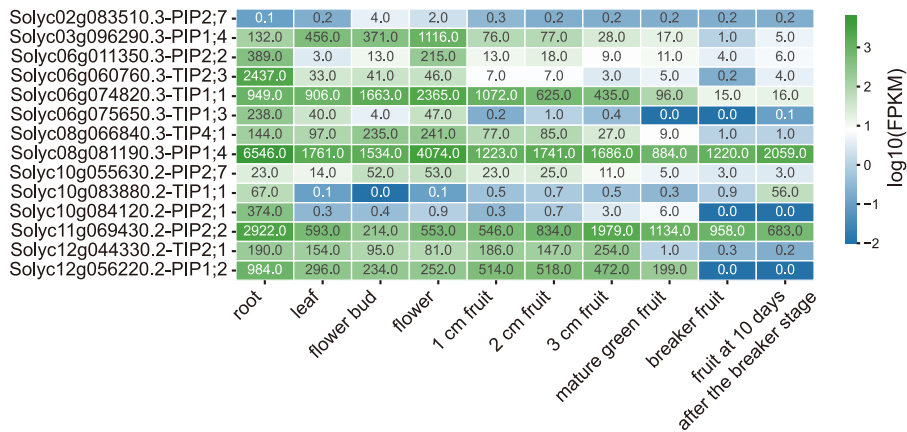

DT

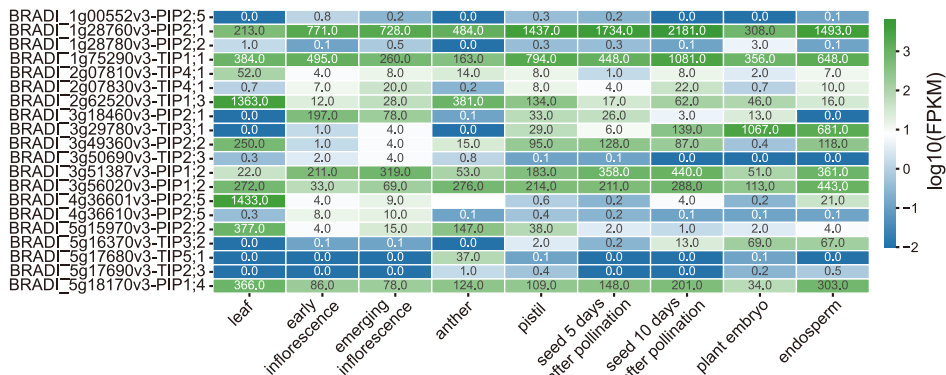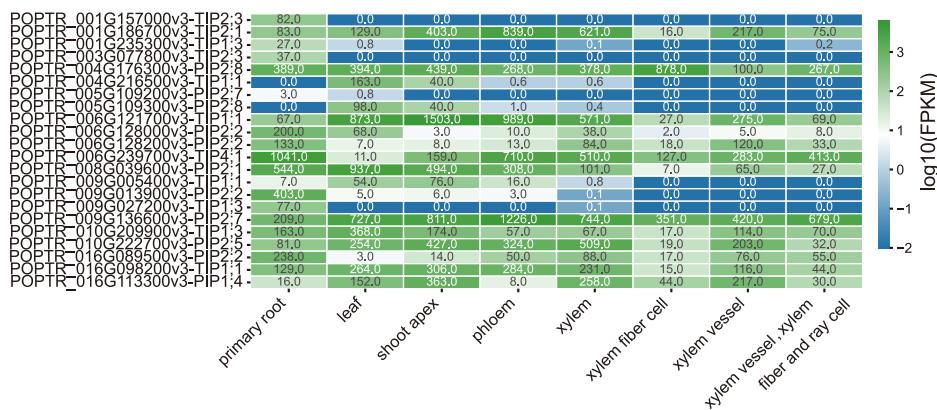

Supplement: Web_Material_uhaf209 [file web_material_uhaf209.zip › Figure S13.pdf]

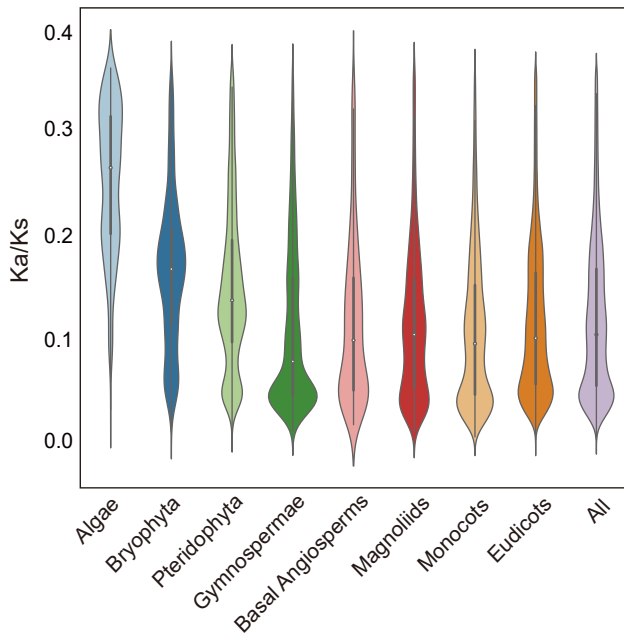

Supplement: Web_Material_uhaf209 [file web_material_uhaf209.zip › Figure S2.pdf]

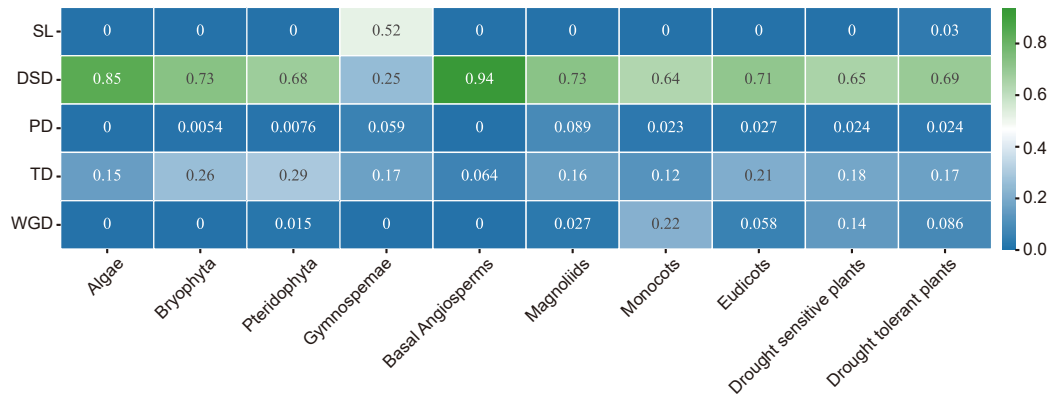

Supplement: Web_Material_uhaf209 [file web_material_uhaf209.zip › Figure S3.pdf]

A

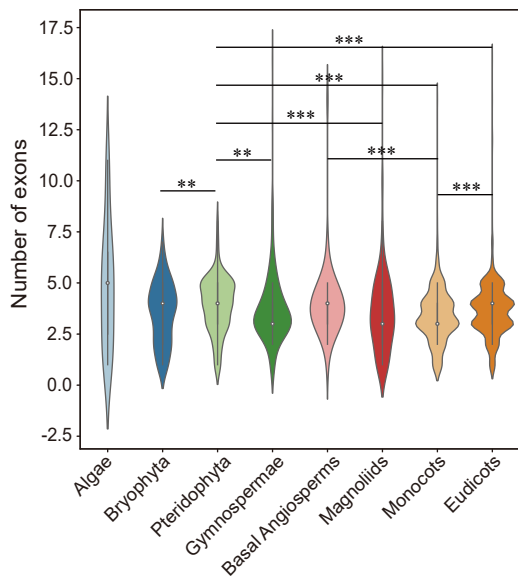

B

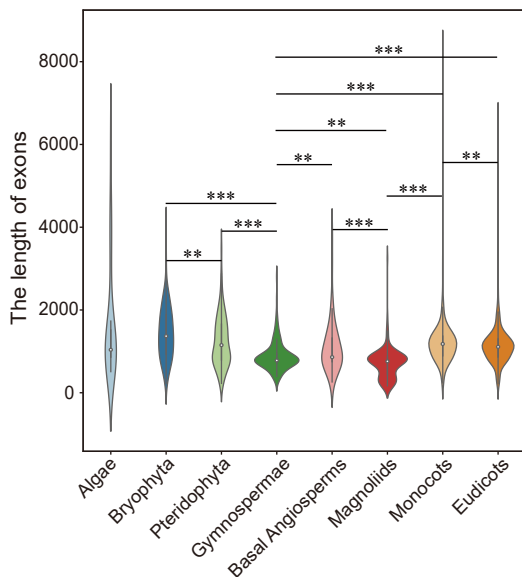

C

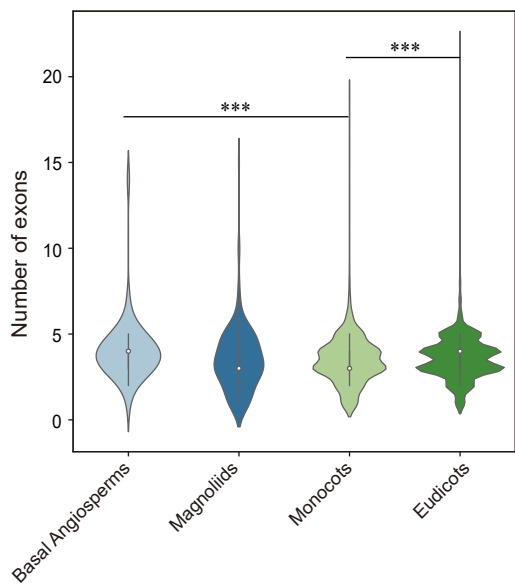

D

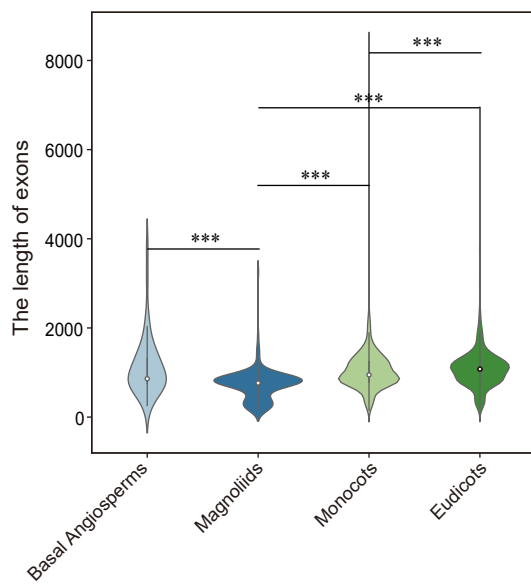

Supplement: Web_Material_uhaf209 [file web_material_uhaf209.zip › Figure S4.pdf]

A

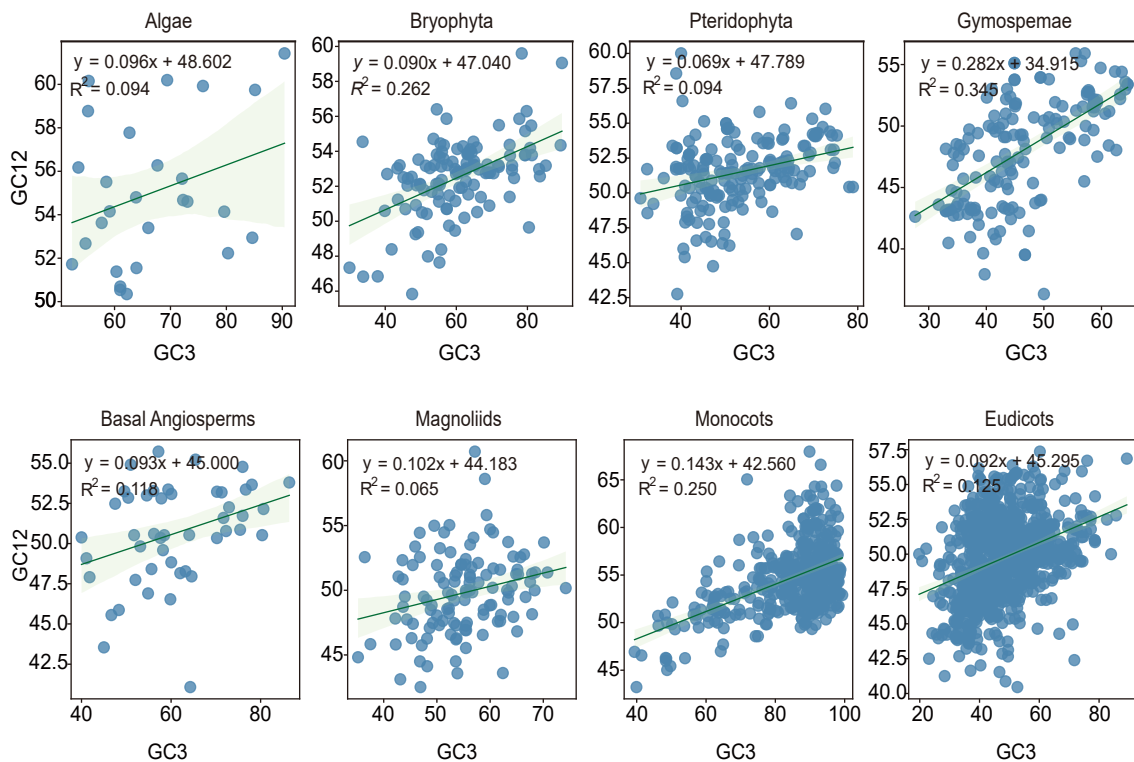

B

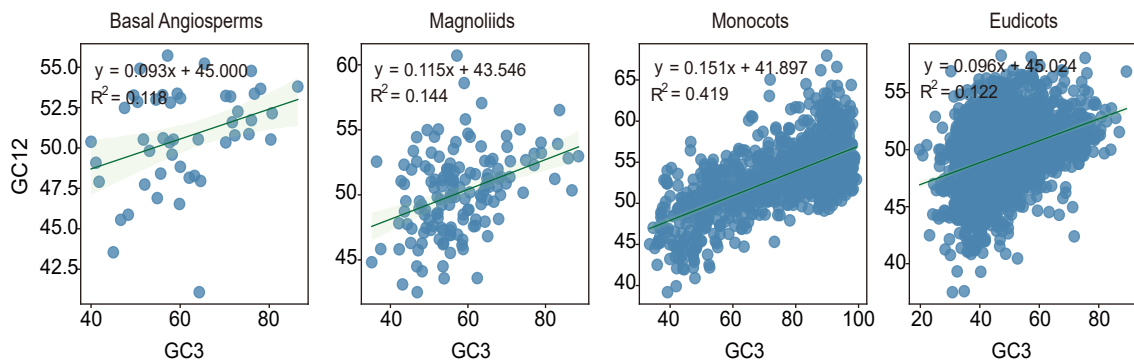

Supplement: Web_Material_uhaf209 [file web_material_uhaf209.zip › Figure S5.pdf]

A

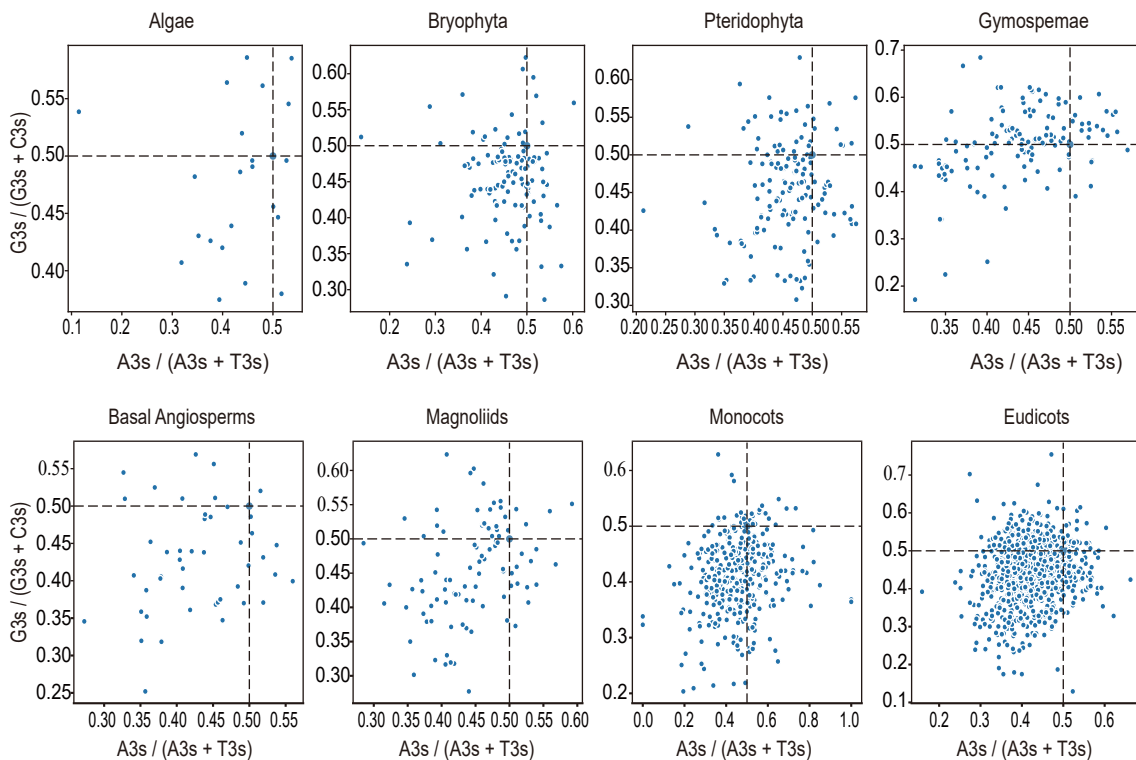

B

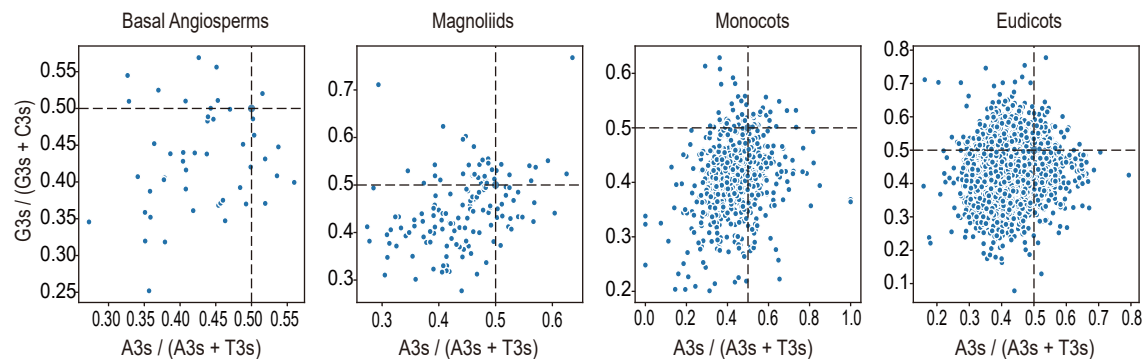

Supplement: Web_Material_uhaf209 [file web_material_uhaf209.zip › Figure S6.pdf]

A

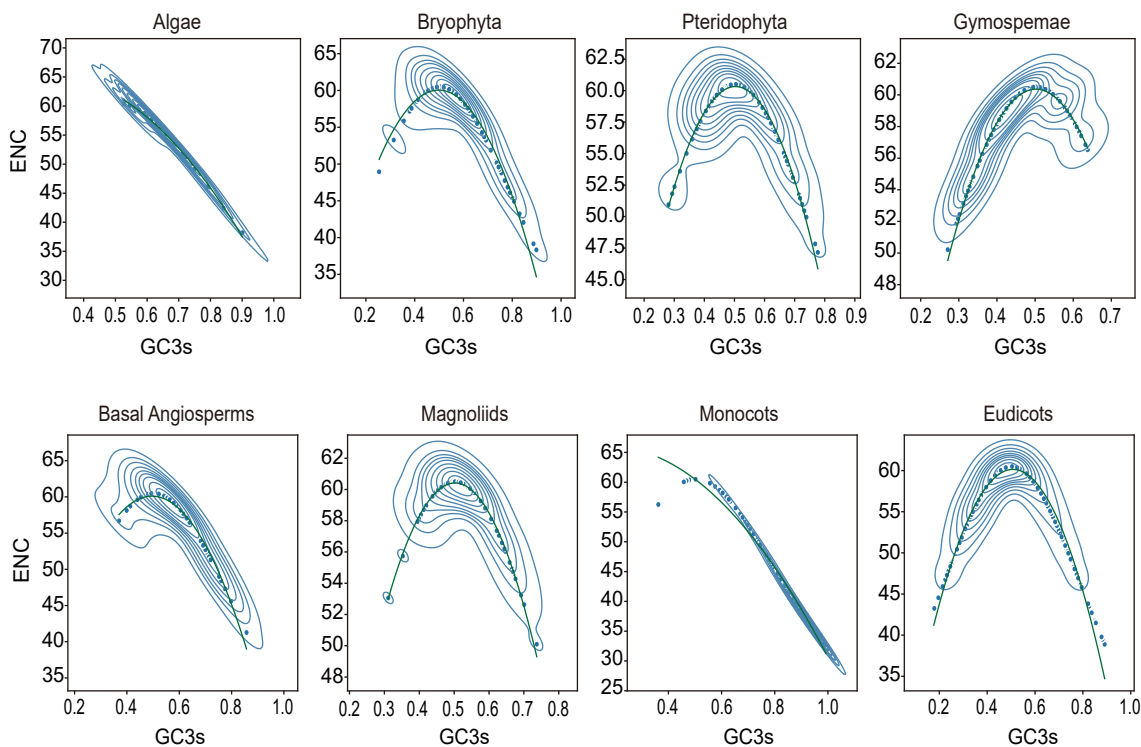

B

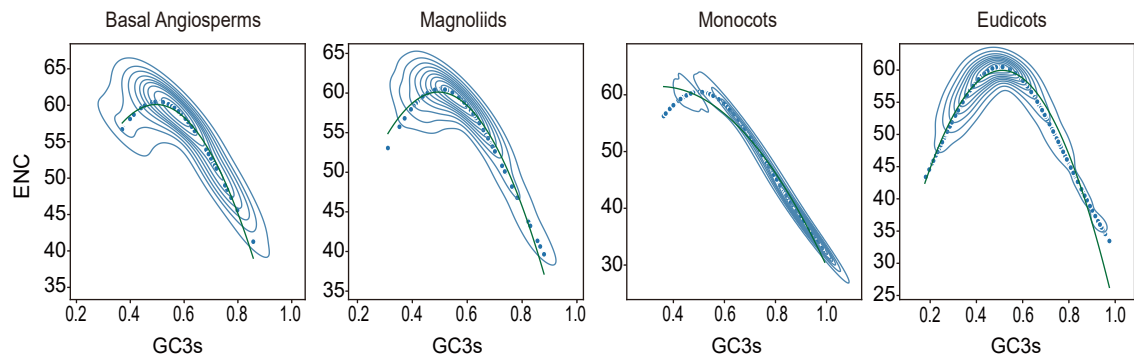

Supplement: Web_Material_uhaf209 [file web_material_uhaf209.zip › Figure S7.pdf]

A

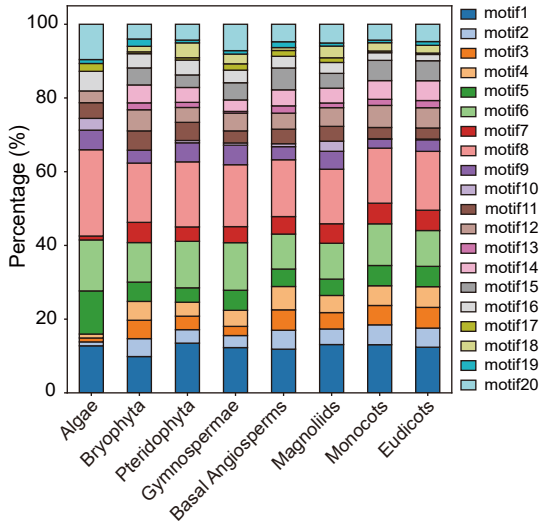

B

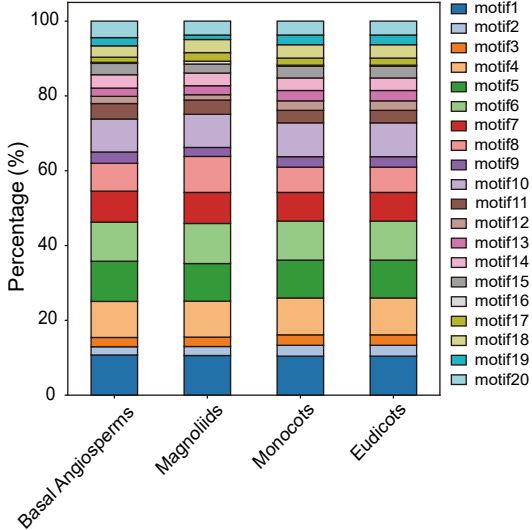

Supplement: Web_Material_uhaf209 [file web_material_uhaf209.zip › Figure S8.pdf]

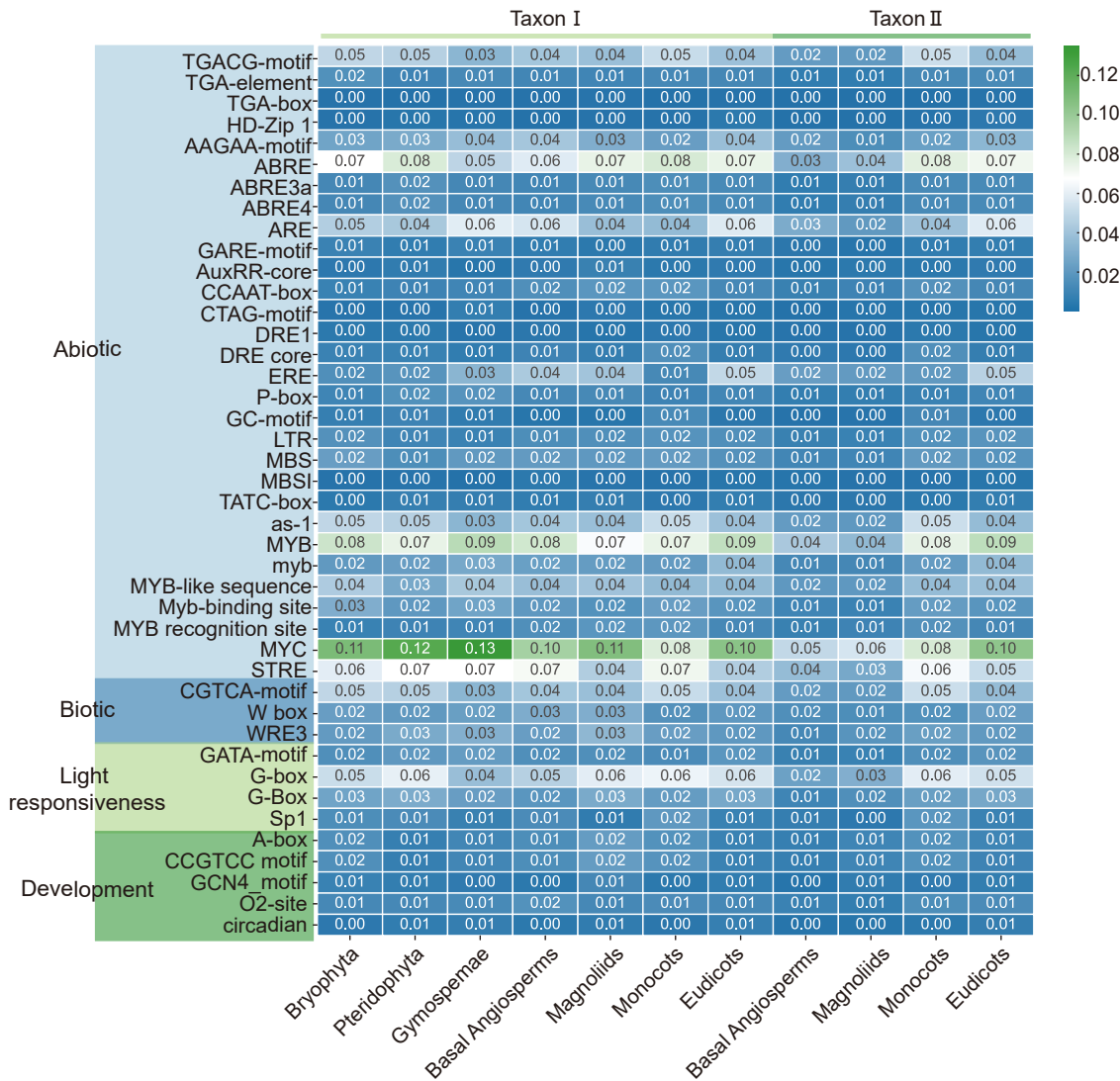

Supplement: Web_Material_uhaf209 [file web_material_uhaf209.zip › Figure S9.pdf]
